# Effects, barriers and facilitators in predischarge home assessments to improve the transition of care from the inpatient care to home in adult patients: an integrative review

**DOI:** 10.1186/s12913-021-06386-4

**Published:** 2021-06-02

**Authors:** Uta Kirchner-Heklau, Kai Krause, Susanne Saal

**Affiliations:** Institute of Health and Nursing Science, Medical Faculty of Martin Luther University, Halle-Wittenberg, Germany

**Keywords:** Patient discharge, Home visit, Systematic review, Occupational therapy, Rehabilitation, Virtual reality

## Abstract

**Background:**

Predischarge home assessments (PDHA) aim to support safe discharge from hospital or rehabilitation. There is insufficient evidence on the effectiveness of PDHA. For adults with any diagnosis, we aimed to determine (1) the effects of PDHA on outcomes associated with the successful return to community living (e.g., Activities of Daily Living, falls) and (2) the associated barriers and facilitators in order to derive recommendations for clinical practice.

**Methods:**

We searched Medline, EMBASE, CINAHL, five additional databases and other sources. We included individual and cluster randomized (RCT/cRCT) and controlled clinical trials comparing PDHA versus usual care/other intervention, as well as qualitative/mixed methods studies dealing with PDHA. Critical appraisal was performed according to the Cochrane risk-of-bias tool in quantitative studies and the Critical Appraisal Skills Programme (CASP) as well as the McMaster University Guidelines for Critical Review Form for qualitative studies and data extraction. Meta-analysis, thematic synthesis and integrative synthesis were performed.

**Results:**

Eight RCTs (*n* = 1072) and ten qualitative studies (*n* = 336) met the inclusion criteria. RCTs reported a variety of outcomes (*n* = 17). We are uncertain if PDHA has any effect on patient outcomes in Activities of Daily Living, quality of life, mobility and fear of falling, falls and hospital readmissions (with moderate to very low quality of the evidence). The qualitative studies revealed facilitators and barriers which should be considered by therapists when conducting PDHA. These were related to the following topics: patient safety education, patient information, patients’ acceptance of modifications and aids, functional assessment, standardization of procedures as well as the consideration of relevant patient conditions and contextual factors in PDHA.

**Conclusion:**

There is no evidence from the meta-analysis for the effectiveness of PDHA. Further robust studies are needed to adapt and evaluate PDHA interventions, taking the identified stakeholders’ views on PDHA into account and following the current recommendations for the development and evaluation of complex interventions.

**Trial registration:**

The review was registered and methods were reported on PROSPERO on 18th July 2018 (CRD42018100636).

**Supplementary Information:**

The online version contains supplementary material available at 10.1186/s12913-021-06386-4.

## Background

Discharge planning aims to support patients’ independence in activities of daily living (ADL) and participation in life and to support a safe home environment to prevent falls and injuries that could lead to hospital readmissions; therefore, predischarge home assessments are an important component of discharge planning.

Predischarge home assessments (PDHA) are conducted while the patients are in hospital or inpatient rehabilitation in order to gain information for therapy and discharge management including the provision of aids and home modifications before the transition to the patient’s home (or nursing home) [1]. The way in which information about the home environment is gathered varies [2–4]. Physical home visits with the patient are described as costly and time consuming [3, 5, 6]. The ward-based collection of environmental information data can be obtained by interviews. These interviews can be supported by the technological visualization of the home environment [2, 3, 7]. Access visits are made to gain information about the patient’s home without the patient being present [2, 8]. Therefore, access visits and all types of ward-based assessments do separately and independently assess both the home environment and the patient’s functioning. An assessment of the patient’s functioning within his/her specific home environment can only be provided during occupational therapy visits when the patient is present. All types of home assessments by occupational therapists aim at preparing and improving the patient’s hospital discharge to his previous or a new residence, respectively and are considered in this study under the term PDHA.

There is limited evidence on the effects of PDHA. A recent systematic review analyzing the effects of predischarge home visits and their influencing factors [4] included five RCTs, one cohort study and three retrospective medical record/chart audits as well as four interview studies and one questionnaire survey. The studies were of low to moderate quality and reported a small decrease in the risk of falling, but no other statistically significant effects.

In recent years, studies on new technologies for PDHA have been published, which were not included in the review by Lockwood et al. [4]. These studies focus on 3-D visualization that offers computer-generated environments, scenarios and objects [2, 3, 9] that can be used to avoid travelling to a patient’s home and to improve the patient’s involvement in home modification planning. Thus, an update of the evidence synthesis on PDHA is warranted.

There is some information about stakeholders’ views on the PDHA process. In their review, Lockwood et al. also investigated the patients’ and carers’ perceptions of PDHA effectiveness and included five qualitative studies, reporting on three emerging themes: *satisfaction with the process*, *purpose of the visit*, *and incorporation of patient and carer opinions in the decision-making process* [4]. The authors concluded that it might have an impact on the effects of the intervention and *how* PDHA are conducted, and recommended consultation and patients’ participation in the PDHA process [4]. A thematic synthesis included five qualitative studies and reported the experiences and perceptions of older adults concerning PDHA. It is required that patients understand the purpose of PDHA and therapists are open-minded towards the coping strategies of older adults [10]. In recent years, a number of qualitative studies investigating the views of stakeholders of the PDHA process have been published.

Therefore, we conducted a mixed methods review aiming to determine the effects of PDHA on outcomes associated with a successful return to community living and to update the evidence on barriers and facilitators in the PDHA process to derive recommendations for improving PDHA.

## Methods

### Protocol and registration

The review was registered in the International Prospective Register of Systematic Reviews (PROSPERO) under the identification number CRD42018100636. The protocol and the review were reported according the recommendations of the Preferred Reporting Items for Systematic Reviews and Meta-Analyses statement (PRISMA) [11] and the framework to enhance transparency in reporting the synthesis of qualitative research (ENTREQ) [12].

### Eligibility criteria

Quantitative clinical trials were included if

1. an individual or cluster-randomized controlled or controlled trial design was used,
2. the study participants were aged ≥18 years, admitted to hospitals or rehabilitation facilities with any diagnosis at all. Studies with participants in psychiatric and perinatologic settings were excluded.
3. PDHA was reported as a primary intervention, though it could vary in purpose (e.g., discharge planning or functional assessment), delivery mode (e.g., with / without a patient), intensity, length and frequency,
4. PDHA was compared to another intervention or to usual care,
5. the outcomes were associated with a successful return to community living (e.g., functioning in the home environment, readmissions), quality of life, patient satisfaction, caregiver burden and / or the immediate output of the predischarge home assessment (e.g., home modifications), and if
6. the study was published in the English or German language.

Qualitative studies and mixed methods studies were included if they reported on views and opinions, perspectives, beliefs, feelings, understanding, experiences or behavior regarding PDHA of adult stakeholders (e.g., patients, healthcare providers) and were published in the English or German language.

### Search strategy and selection criteria

In July 2018, Medline, EMBASE, CINAHL, the Cochrane Central Register of Controlled Trials (CENTRAL) PEDro and OTseeker databases and trial registries (PROSPERO and ICTRP) were searched. The overall search strategy used the following combined search terms for database searches: discharge, inpatient, sub-acute care, acute care, rehabilitation [MeSH], House Calls [Mesh], home visiting, home visit, environmental assessment, assessment visit, home safety, home modification, environmental modification, weekend passes, weekend pass (see Additional file 1 for search strategy). Relevant RCTs, CCTs and qualitative studies were included in the analysis. References of the identified publications were checked from July 2018 until December 2018. A literature update was undertaken between the 9th and 14th of September 2020. Forward citation search was conducted using Google Scholar and Web of Science.

### Data collection and analysis

#### Study selection

Two independent reviewers (KK, UKH) applied the inclusion and exclusion criteria to titles and abstracts of the search results. Discrepancies were discussed and resolved by consensus with a third author (SuS) and by reading the full text, if needed. The remaining sample of studies was read in full text by two independent reviewers (KK, UKH). Discrepancies were discussed and resolved by consensus with a third author (SuS). Inclusion was unclear in one case (Gursen, 2003) due to insufficient reporting on the study design. After trying to contact the authors without any success, we excluded the study. Multiple publications reporting on the same study were clustered and handled as one unit. See Additional file 1 for excluded studies.

#### Data extraction and management

One reviewer extracted the descriptive information from the publications using a piloted data extraction sheet, and another reviewer double-checked the extracted data. The following information was extracted for quantitative and qualitative studies: aim and focus of the studies, study design, details about the intervention according to the TiDier Checklist [13], number and characteristics of participants, outcomes, and outcome measures. For quantitative studies, raw scores were extracted using Excel sheets. If outcomes were measured at multiple time points, the latest follow-up was selected. If studies reported on outcomes using more than one measure, we used only one measure per outcome, according to a pre-specified hierarchy, determined by the researcher group (see Additional file 2). For qualitative studies, we extracted verbatim quotes from study participants and the authors’ descriptions of the findings from the results section.

#### Risk of bias assessment

Two reviewers (KK, UKH) independently assessed the risk of bias. Any disagreements were resolved by discussion and, if necessary, by consulting a third author (SuS). We used the methods and recommendations for the assessment of risk of bias and heterogeneity in individual quantitative studies as described in the Cochrane Handbook 5.1.0 [14].

For qualitative studies, a set of criteria from the CASP tool [15] as well as from the Guidelines for Critical Review Form: Qualitative Studies, Version 2 [16] was used to assess the internal validity (see Additional file 4 for quality appraisal of qualitative studies).

#### Data analysis and synthesis

In case only median, sample size and interquartile range (with first and third interquartile) were presented and imputing SDs was not possible, we estimated the sample mean and standard deviation according to Wan et al. [17]. Meta-analysis was conducted using a random-effects model (REM). We decided to use and report the effects in the fixed effect model additionally, if the heterogeneity was rather low, since the random effects model makes some assumptions of its own about the distribution of the study effects, which may not be accurate due to the small number of studies [14]. For continuous outcome data, we used standardized mean differences (SMD) with 95% confidence intervals (CIs) for different scales or units and mean differences (MD) with standard deviations (SD) for same scales. For meta-analysis in dichotomous data, we calculated risk ratios (RR), which were defined as the average number of events per participant, with 95% CIs. We used the I^2^ test for the assessment of statistical heterogeneity, a significance level of p less than 0.10, and the chi-squared test.

We assumed that effect sizes may differ due to different scales per outcome and conducted a corresponding sensitivity analysis. Due to the small number of studies that differed per pooled outcome in several aspects (diagnosis, type of intervention, time of measurement) at the same time, we decided to forgo a post-hoc subgroup analysis, according to the recommendations of the Cochrane Handbook for Systematic Reviews of Interventions Version 5 [18]. To judge the quality of the evidence, the GRADE approach [19] was used. See Additional file 5 for details on our GRADE ratings.

Qualitative data were entered verbatim using MAXQDA 2018.2 software for data analysis. Thematic analysis was applied [20]. Line-by-line coding was performed by two reviewers independently to reconcile comprehension. The descriptive themes were discussed iteratively with the whole team (KK, UKH, and SuS) until a consensus regarding comprehensibility and distinction of themes was reached. We synthesized findings according to the emerging themes related to barriers and facilitators of the PDHA process. Implications for practice and intervention development were inferred, according to the methods for thematic synthesis by Thomas, [21]. Based on the facilitating factors and barriers in certain subject areas, we elaborated how a PDHA intervention should be designed in order to take into account the views of patients and healthcare professionals.

An integrative synthesis of quantitative results and implications from qualitative studies was performed, whereby two reviewers (KK, UKH) examined the intervention descriptions of the included RCTs to identify whether implications were addressed or not. The detailed information supporting the decisions was discussed and documented. A matrix of the integrative synthesis mapped the studies’ effect sizes with contextual details and information on corresponding implications and interventions (please see Additional file 8).

## Results

### Study selection

**Fig. 1:**
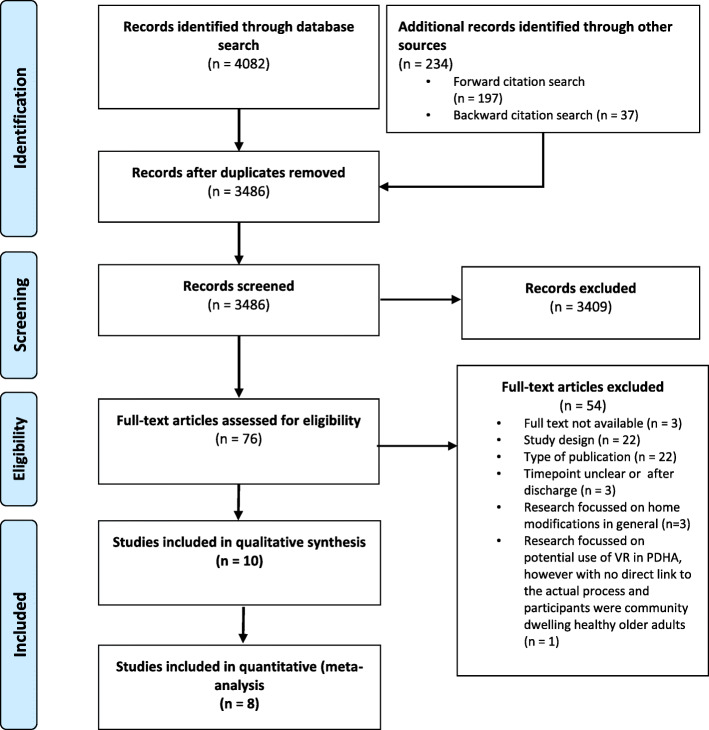
Flow diagram of study selection

Our search revealed 3486 publications (Fig. 1), of which 22 publications met our inclusion criteria. These publications referred to eight RCTs and ten qualitative studies.

### Study characteristics

Eight RCTs with 1149 participants were included [1, 2, 8, 22–24]. Three additional publications referred to these original RCTs [25–28]. The size of the studies ranged from ten to 400 participants. The cohort study alongside one RCT was not considered in the analysis [8].

Ten qualitative studies with a total of 323 participants (range: *n* = 4 [29] to *n* = 122 [30]) were included [3, 29, 31–36]. All the studies used interview techniques, one used participant observation additionally [35], and another one used a semistructured survey [30]. Two studies explored perceptions of patients and therapists with regard to the use of virtual reality (VR) applications in PDHA [3, 32]. Three studies explored factors considered by OTs when deciding about stroke patients’ need for a predischarge home assessment visit, as well as clinical reasoning and practice of PDHA [30, 34, 36, 37]. One study focused on older adults’ and carers’ perception of and involvement in PDHA decision-making processes [31]. Another study also highlighted the patients’ perspective on PDHA [29]. A summary of characteristics of the included quantitative and qualitative studies is displayed in Table 1.

**Table 1:** Study characteristics of included studies

Reference	Study design, country, setting	Participants Number, age in years, percent female (♀%)	Intervention description	Outcomes/aim of qualitative research	Outcome measures	FU in mo nths
Clemson et al., 2016 [7]	RCT Australia Acute care, unspecified	*n* = 400 **Intervention** 80.2 (±6.4, range n.r.) ♀ 59.6% **Control** 80.7 (± 5.7, range n.r.) ♀ 63.9%	**Intervention** In-hospital rapport building, interview, ADL-assessment, predischarge home visit, post-discharge home-visit, telephone calls **Control** Usual care, in-hospital interview, ADL assessment, access visit if required	Functional independence, participation in ADL, unplanned readmissions, emergency department visits, recommendations	NEADL [38], Late Life Disability Index (LLDI) - sub scores: frequency and limitation [39], number of: recommendations, unplanned readmissions, emergency department visits, falls, process outcomes (e.g., number of prescribed and tried equipment; effects not estimated)	3
Drummond et al., 2013 [8]	RCT UK Stroke rehabilitation unit	*n* = 126 **Intervention** 70.64 (± 14.29, range 34–88) ♀ 54.7% **Control** 73.65 (± 16.06, range 41–99) ♀ 47.8%	**Intervention** One or two predischarge home visits **Control** Structured home assessment interview	ADL/IADL, mobility, unplanned readmissions, falls, emotional distress in medical settings, depressed mood of clients with stroke and significant aphasia, caregiver strain	NEADL [38]*,* Barthel Index [40], RMI [41], Number of unplanned readmissions, GHQ-28 [42], SADQ-10 [43], Caregiver Strain Index [44]	1
Hagsten et al., 2004 [22] Hagsten et al., 2006 [25]	RCT Sweden Acute care, hip fractures	*n* = 100 **Intervention** 81 (± 23, range 68–91) ♀ 84% **Control** 79 (± 30, range 65–95) ♀ 76%	**Intervention** Individual daily training, including use of technical aids, single predischarge home visit **Control** One session of walking instruction when in hospital	ADL/IADL, health-related quality of life	ADL [45], EQ. 5D [46], IADL single scales: moving around indoors; performance of light housework; getting in and out of a car, SWED-QUAL [47] subscales	2
Lannin et al., 2007 [23]	RCT Australia Rehabilitation unit, mixed (cardiac, orthopedic trauma, neurological, orthopedic joint surgery, spinal or deconditioned)	*n* = 10 **Intervention** 80.0 (± 7, range n.r.) ♀ 100% **Control** 82.4 (± 7, range n.r.) ♀ 60%	**Intervention** Single predischarge home visit **Control** In-hospital consultation prior to discharge	ADL/IADL, mobility, unplanned readmissions, falls, fear of falling, community support, health-related quality of life	NEADL [38], FIM [48], RNLI - Reintegration to Normal Living Index [49], Tinnetti [50], number of unplanned readmissions, number of falls, FES-I [51], EQ. 5D [46], EQ-5D VAS [52]	7
Lockwood et al., 2019 [53]	RCT Australia Acute and rehabilitation, hip fractures	*n* = 77 **Intervention** 83.4 (± 7.1, range n.r.) ♀ 76% **Control** 80.9 (± 7.3, range n.r.) ♀ 68%	**Intervention** Single predischarge home visit and usual care **Control** usual care	ADL/IADL, health-related quality of life, unplanned readmissions, number of days in hospital after index discharge, falls, fear of falling, adverse events	NEADL [38], SMAF [54], FIM [55], FES-I [51], EQ. 5D [46], EQ-5D VAS [52]	6
Lockwood et al., 2020 [26] ^b^	RCT, process evaluation Australia Acute and rehabilitation, hip fractures	n = 77 **Intervention** 83.4 (± 7.1, range n.r.) ♀ 76% **Control** 80.9 (± 7.3, range n.r.) ♀ 68%	**Intervention** Single predischarge home visit and usual care **Control** usual care	Recommendations, adherence to recommendations	Number of recommendations, Number of adherence to recommendations	1
Nikolaus et al., 2003 [1]	RCT Germany Geriatric acute care, unspecified	*n* = 360 **Intervention** 81.2 (±6.2, range 84.9–87.5) ♀ 72.4% **Control** 81.9 (±6.5, range 74.4–88.4) ♀ 74.3%	**Intervention** Predischarge home visit and post-discharge follow-up visit(s), comprehensive in-hospital geriatric assessment **Control** Comprehensive geriatric assessment and usual care	Falls, recommendations	Number of falls, compliance with recommendations after 12 months	12
Pardessus et al., 2002 [24]	RCT France Geriatric acute care, unspecified	*n* = 60 **Intervention** 83.51 (± 9.08, range n.r.) ♀ 76% **Control** 82.9 (± 6.33, range n.r.) ♀ 80%	**Intervention** Predischarge home visit **Control** Usual care	ADL/IADL, Falls, re-hospitalization, institutionalization	IADL [56], SMAF subscales [54] ADL subscales [57] number of recurring falls, mean number of fall recurrence in former fallers, number of re-hospitalizations, number of institutionalizations	12
Provencher et al., 2020 [27] ^a^	RCT, Post-hoc analysis Australia Acute care, unspecified	*n* = 400 **Intervention** 80.2 (±6.4, range n.r.) ♀ 59.6% **Control** 80.7 (± 5.7, range n.r.) ♀ 63.9%	**Intervention** In-hospital rapport building, interview, ADL-assessment, predischarge home visit, post-discharge home visit, telephone calls **Control** Usual care, in-hospital interview, ADL assessment, access visit if required	Functional independence, participation in ADL, unplanned readmissions, emergency department visits, recommendations	NEADL [34], Late Life Disability Index (LLDI) - sub scores: frequency and limitation [58], number of: recommendations, unplanned readmissions, emergency department visits, falls, process outcomes (e.g., number of prescribed and tried equipment; effects not estimated)	3
Threapleton et al., 2018 [2]	RCT UK Stroke ward, acute care	*n* = 16 **Intervention** 72 (±21.08, range 38–90) ♀ 75% **Control** 70 (± 12.6, range 46–86) ♀ 37%	**Intervention** Single predischarge virtual home assessment **Control** Usual care	ADL/IADL, overall independence, mobility, fear of falling, health-related quality of life	NEADL [38], Barthel- Index [40], MRS [59], Rivermead Mobility Index [41], FES-I [51], EQ. 5D [46]	6
Wales et al., 2018 [28] ^a^	RCT, economic evaluation Australia Acute care, unspecified	*n* = 400 **Intervention** 80.2 (±6.4, range n.r.) ♀ 59.6% **Control** 80.7 (± 5.7, range n.r.) ♀ 63.9%	**Intervention** In-hospital rapport building, interview, ADL-assessment, predischarge home visit, post-discharge home visit, telephone calls **Control** Usual care, in-hospital interview, ADL assessment, access visit if required	Costs for predischarge home visits	costs for occupational therapy time, travel, community follow up, hospital readmission	3
Atwal et al., 2008 [31]	Semi-structured interview UK Geriatric acute care	**Patients, main carers** *n* = 15 86,46 years (range 73–97) ♀ 60%	**Intervention** Single predischarge home visit	- To explore older adults’ and carers’ involvement in decisions that were made during the home visit; - To explore older adults’ and carers’ perceptions of the home visit process	n.a.	n.a.
Atwal et al., 2014a [32]	Semi-structured interview; think aloud technique UK Acute care and community care	**OTs** *n* = 7 ♀ 71% social services, older persons, mental health, acute care, pediatrics	**Intervention** Virtual reality predischarge home assessment with interior design application	- To explore occupational therapists’ perceptions of a virtual reality interior design application (VRIDA); - to gain insights into the feasibility of using VRIDA as a tool to aid the predischarge home visit (perceived usefulness, perceived ease of use, actual use)	n.a.	n.a.
Atwal et al., 2014b [37]	Semi-structured interview UK Acute care, intermediate care, rehabilitation, older patients, mental health (older people)	**OTs** *n* = 21	**Intervention** Predischarge home visit / access visit	- To explore occupational therapists’ perceptions of home visits; - To ascertain occupational therapists’ clinical reasoning with respect to conducting home visits	n.a.	n.a.
Cameron et al., 2014 [33]	In-depth interview, semi-structured, focus groups Canada Rehabilitation facility, stroke	**Patients** *n* = 16 62 years (range 25–87) ♀ 75% **Family caregivers** n = 15 41 years (range 23–75) ♀ 86,7% **Multiple health professionals** *n* = 20	**Intervention** Single predischarge home visit or preparation in hospital and single/multiple predischarge weekend passes	- To explore stroke survivors’, caregivers’, and healthcare professionals’ perceptions of weekend passes offered during inpatient rehabilitation and its role in facilitating the transition home	n.a.	n.a.
Davis et al., 2019 [30]	Semi-structured survey Republic of Ireland acute settings, rehabilitation settings and convalescence settings, adult patients (over 18 years)	**OTs** *n* = 122	**Intervention** Pre-discharge home visit	- To investigate clinical practice during DPHV and the clinical reasoning guiding occupational therapists within an Irish context	n.a. for semistructured part quantitative part of survey: use of standardized tool, contents of home visit bag, numbers of recommendations, consensus on clinical practice	n.a.
Godfrey et al., 2019 [34]	Focus-group interviews Australia Acute or sub-acute settings from three facilities	**OTs** *n* = 19 **Multidisciplinary stakeholders** *n* = 8	**Intervention** Pre-discharge home visit	- To understand both occupational therapists’ and multidisciplinary stakeholders’ perceptions and contemporary practice regarding decision-making and pre-discharge home visits through exploration of experience and current practice in the Australian context. Investigation of factors associated with when, how and to whom pre-discharge home visits are provided	n.a.	
Hibberd, 2008 [29]	Semi-structured interview UK Intermediate care unit	**Patients** *n* = 4 65 years and older ♀ 50%	**Intervention** Predischarge home visit / access visit	Part of an evaluation study; - To gain patient perspectives on home visiting process -to ensure service meets needs	n.a.	n.a.
Nygard et al., 2004 [35]	Interviews, focus groups, participant observation Sweden Geriatric acute care, mixed diagnoses	**Patients** *n* = 23 78 years (range 68–86) ♀ 50% **Living alone** *n* = 12 **OTs** *n* = 9	**Intervention** Single predischarge home visit	- To describe and illustrate, from both clients’ and therapists’ perspectives, the occupational therapy interventions and recommendations that were undertaken and followed-up in common practice during predischarge home visits; - To gain insight in the accuracy of expectations of therapists and in perceived usefulness of predischarge home visits to clients	n.a.	n.a.
Threapleton et al., 2017 [3]	Semi-structured interview UK Acute care, rehabilitation, community, stroke	**Patients** *n* = 8 68 years (range 44–92) ♀ 75% **Stroke survivors** ***n*** = 4 70 years (range 61–79) ♀ 75% **OTs** *n* = 13	**Intervention** Virtual predischarge home visit	- To explore perceptions concerning the acceptability, potential utility and limitations of the use of a virtual reality interior design application from the perspectives of therapists and patients	n.a.	n.a.
Whitehead et al., 2014 [36]	Semi-structured interview UK Acute, rehabilitation, mixed, hyper acute, stroke	**OTs** *n* = 20	**Intervention** Predischarge home assessment visits	- To explore what factors occupational therapists consider when deciding which patients with a stroke need a predischarge home assessment visit	n.a.	n.a.

*FU* latest time point of follow-up, *n.a* Not applicable, *n.r.* Not reported, ^a^ refers to the original RCT of Clemson et al., 2016 [7]; ^b^ refers to the original RCT of Lockwood et al., 2019 [53]

#### Setting and participants

The studies were published between 2002 and 2020, and the majority were conducted in the UK [2, 3, 8, 29–32, 36, 37] and Australia [7, 23, 26–28, 34, 53, 60]. One study each was carried out in Germany [1], France [24] and Canada [33], and two studies were conducted in Sweden [22, 25, 35].

Participants in RCTs were recruited in acute care settings [1, 2, 7, 22, 24–28, 53] and rehabilitation units [8, 23], and for qualitative studies in rehabilitation [33], in acute care [3, 31] and in intermediate care [29].

Diagnoses were mixed, not specified or not sufficiently reported in five RCTs [1, 7, 23, 24, 28] and in seven qualitative studies [29–32, 34, 35, 37]. In two RCTs and three qualitative studies, participants had suffered from a stroke [2, 3, 8, 33, 36]. The diagnosis was hip fracture in twoRCTs [22, 25, 26, 53].

The qualitative studies reported on participants’ views [3, 29, 33, 35, 60] and on views of OTs [3, 31–33, 35, 36] or families [33, 60].

#### Types of interventions

Interventions comprised a single predischarge home visit only [2, 8, 23, 53] as well as additional supportive interventions through in-hospital activities [1, 22, 24, 25], including extended assessment [1, 7, 23] and / or extended training [1, 25]. Further intervention components were patient education [2, 8, 23, 24] and post-discharge follow-ups [1, 7]. All the PDHAs were conducted by OTs alone, or with additional professionals allied to health care (physiotherapists, nurses, social workers) [1, 24]. The patients were present during the home assessment in seven out of eight RCTs [2, 7, 8, 22–25, 53]. All but one of the interventions were conducted in the patient’s home, and included functional assessment [1]. Virtual home visits, conducted at the hospital, were investigated in one study [2]. The intervention details are available from the corresponding author.

#### Types of comparators

Usual care in Australia was described as an in-hospital access to multidisciplinary care [53], as well as a structured interview with the OT, including two structured assessments and an access visit if more information was required, such as measurements for rails [7] or additional patient education and information about equipment use and community services [23]. Usual care in the UK was described as structured interviews and general discussions about potential problems, and referring to agencies [8]. One study [2] reported additional home / access visits as a control, if required. Usual care in Sweden [22, 25] comprised nursing care and instruction from a physiotherapist for walking aids. Usual care in Germany [1] comprised comprehensive geriatric assessment and recommendations. Usual care in France was not described [24].

#### Risk of bias within studies

**Fig. 2:**
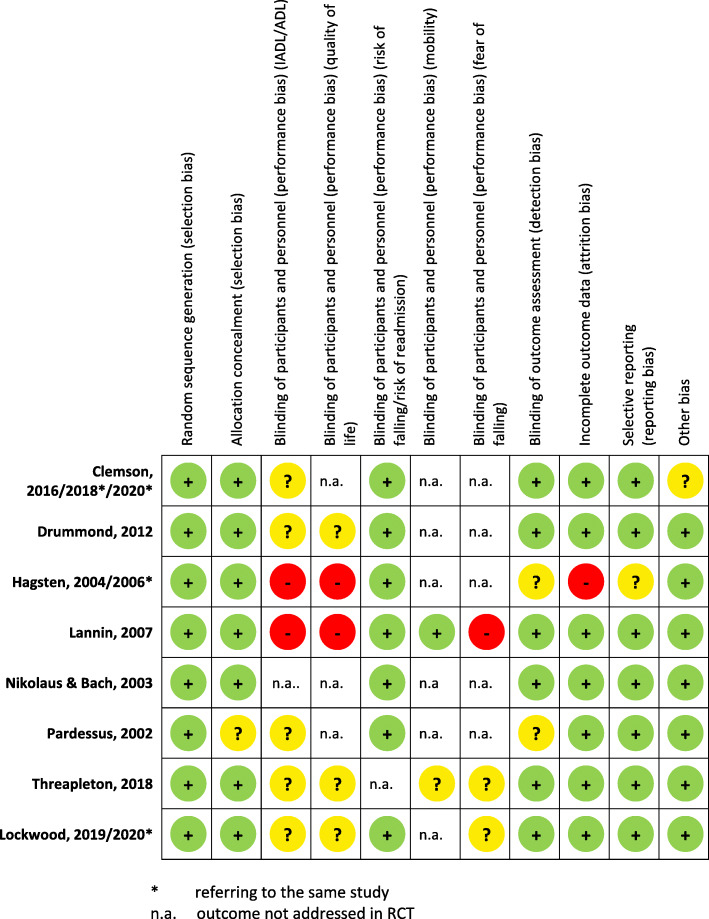
Risk of bias in single studies

The results of the risk of bias assessment are summarized in Fig. 2 and are presented in more detail in Additional file 3. Risk of selection bias was low in all but one study, where it was unclear [24]. For the outcome IADL/ADL, the risk of performance bias was unclear in five studies [2, 7, 8, 24, 53], and high in two of the seven studies addressing this outcome. For quality of life, the risk of performance bias was high in two studies [22, 23, 25] and unclear in three of five studies addressing this outcome [2, 8]. Risk of readmission and risk of falling were not biased in all six studies addressing this outcome [1, 7, 8, 22–25, 53]. Mobility was detected in two studies with a low or unclear performance bias, respectively. Three studies assessed fear of falling with a high or unclear risk of bias, respectively [2, 23, 24, 53]. Risk of detection bias was unclear in two studies [22, 24, 25]. Risk of attrition bias was high in one study [22, 25]. Risk of other bias was unclear in one study [7].

The quality appraisal of the qualitative studies is shown in Additional file 4. The quality of the studies did not influence the analysis since all the studies were considered as being valuable for our research question.

### Effectiveness of PDHA versus usual care

Eight RCTs including 1149 participants compared PDHA with usual care [1, 2, 7, 8, 22–25, 53]. Forest plots for comparisons are displayed in Additional file 6. Meta-analysis was performed for Instrumental Activities of Daily Living (IADL) and Activities of Daily Living (ADL), quality of life (Qol), mobility, fear of falling, risk of falling and risk of readmission. Details on the GRADE judgment are reported in Additional file 5.

The summary of findings for the main outcomes is presented in Table 2.

**Table 2:** Summary of findings

***PDHA compared with usual care for adults with any diagnosis at all***				
***Patients or population****: adults with any diagnosis at all (except mental disorders only)* ***Setting:*** *acute / sub-acute hospital care or rehabilitation unit* ***Intervention:*** *predischarge home assessment* ***Comparison:*** *usual care*				
Outcomes	SMD or MD or RR, [95% CI], I^**2**^, p	Number of participants (number of studies)	GRADE	Comments
***IADL/ADL.*** **Various scales.** Including studies with NEADL, NEADL (60), SMAF, SWED-QUAL Subscale Physical function. Higher score indicates better function. Mean duration of follow-up: 8 months (range 1–12 months)	***SMD − 0.17 [− 0.76, 0.42], I***^***2***^ ***= 90%*** ***p = 0.58***	***655 (7)***	***⨁OOO very low***^***a***^	
***IADL/ADL.*** **NEADL** Score 0–22 points. Higher score indicates better results. Mean duration of follow-up: 2.8 months (range 1–6 months)	***MD − 0.35 [− 1.31, 0.61], I***^***2***^ ***= 79%*** ***p = 0.34***	***510 (5)***	***⨁OOO very low***^***c***^	
***Quality of life.*** **Various scales**: EQ-5D overall ***score,*** EQ-5D subscale VAS, SWED-QUAL subscale general health perception. Higher score indicates better health status. Mean duration of follow-up: 2.6 months (range 1–6 months)	***SMD 0.06 [− 030, 0.42] I***^***2***^ ***= 42%*** ***p = 0.74***	***263 (5)***	***⨁OOO low***^***c***^	
***Quality of life.*** **EQ-5D** overall score 0–1. Higher score indicates better health status. Mean duration of follow-up: 2.6 months (range 1–6 months)	***MD 0.03 [− 0.08, 0.15], I***^***2***^ ***= 0%*** ***p = 0.56***	***186 (3)***	***⨁⨁OO low***^***b***^	
***Mobility.*** **Various scales**: Tinetti (scale 4–24) and RMI (0–15). Higher scores indicate better mobility. Mean duration of follow-up: 2 months (range 1–3 months)	***SMD 1.24 [‘-0.69, 3.17], I***^***2***^ ***= 78%*** ***p = 0.21***	***26 (2)***	***⨁OOO very low***^***b***^	
***Fear of falling.*** **FES-I** Score 10–100. Higher scores indicate more confidence. Mean duration of follow-up: 3.3 months (range 1–6 months)	***MD − 4.01 [− 10.4, 2.05], I***^***2***^ ***= 51%*** ***p = 0.19***	***85 (3)***	***⨁OOO very low***^***c***^	Fixed effect model: (MD − 4.74 [− 8.30, − 1.18] I2 = 51%, *p* = 0.009
***Risk of falling*** Mean duration of follow-up: 9.2 months (range 1–12 months)	***RR 0.88 [0.70, 1.09], I***^***2***^ ***= 0%*** ***p = 0.25***	***501 (5)***	***⨁⨁⨁O***^***d***^ ***moderate***	
***Risk of readmission:*** Mean duration of follow-up: 5 months (range 1–12 months)	***RR 1.09 [0.64, 1.87], I***^***2***^ ***= 43%*** ***p = 0.74***	***590 (5)***	***⨁⨁⨁O***^***d***^ ***moderate***	
***Adverse effects of intervention:***	*Zero adverse events in both groups were reported in one study.*	**59 (1)**		

^a^ downgraded due to unblinded personnel and participants, inconsistency and imprecision of results

^b^ downgraded due to inconsistency and high imprecision of results

^c^ downgraded due to downgrade because of unblinded participants and personnel, and imprecision of results

^d^ downgraded due to imprecision of results; *FE* Fixed effect model, *RE* Random effects model

Assessment of reporting bias through funnel plot analysis was not appropriate due to the small number of studies.

**IADL/ADL** (Instrumental) Activities of Daily Living (IADL/ADL) were measured in seven of eight studies on patients with stroke, hip fractures, or mixed or unspecified diagnoses respectively [2, 7, 8, 22–25, 53]. Five studies used the Extended Activities of Daily Living scale (NEADL) [38], another used the Functional Autonomy Measurement System (SMAF) [54], each as a full questionnaire. One study used the subscale Physical Function from the Swedish Health-Related Quality of Life Survey (SWED-QUAL), which assesses a patient’s ADL performance (e.g. dressing, climbing stairs) and is therefore comparable to the content of included ADL-measures [47]. There was no overall effect in (instrumental) functions of daily living for participants at the latest follow-up after receiving PDHA when measured with various scales (655 participants, SMD -0.17, 95% CI [− 0.87 to 0.53], *p* = 0.64, I^2^ = 91%). The quality of evidence was judged to be very low due to concerns about risk of bias (blinding of outcome assessment), inconsistency and imprecision with considerable heterogeneity. A sensitivity analysis of five studies using the same scale (NEADL) confirmed the results (MD -0.32 [− 1.26 to 0.61], *p* = 0.50, I^2^ = 0%) with very low heterogeneity [2, 7, 8]. GRADE assessment indicated low quality due to high risk of bias (blinding of outcome assessment) and imprecision.

**Quality of life (QoL)** Three studies used the EQ-5D overall score [52] and another three the subscales of the EQ-5D measure of health status from the EuroQol Group (EQ-5D) or SWED-QUAL [47], respectively. Pooling all studies with any Qol measure [2, 22, 23, 25] showed no statistically significant group differences of PDHA compared to usual care for patients with stroke, hip fractures, or mixed diagnoses respectively, with moderate heterogeneity (263 participants, SMD 0.06, 95% CI [− 0.30 to 0.42], *p* = 0.74, I^2^ = 42%). Applying the GRADE approach, we assessed the quality of the evidence to be very low due to a risk of bias (unblinded participants and personnel) and imprecision of results. A sensitivity analysis of three studies using the same scale (EQ-5D overall score) did not significantly affect the Qol outcome (186 participants MD 0.03, 95% CI [− 0.08 to 0.15], *p* = 0.56, I^2^ = 0%). The quality of the evidence for these results was low due to inconsistency and imprecision.

**Mobility.** Two studies assessed mobility through Performance-Oriented Assessment of Mobility Problems (Tinetti) or The Rivermead Mobility Index (RMI) rating scale for patients with mixed diagnoses or stroke, respectively [2, 23] Pooling these studies showed no improvement at the latest time points of follow-up at one and three months (26 participants, SMD 1.24, 95% CI [− 0.69 to 3.17], *p* = 0.21, I2 = 78%). However, the quality of the evidence was rated very low due to inconsistency and high imprecision based on a very small number of participants with high heterogeneity.

Three studies measured **fear of falling** in participants with a stroke or mixed diagnoses, respectively, using the Falls Efficacy Scale - International (FES-I) [51]. There might be a slight trend towards an increase in fear of falling in participants who received the PDHA intervention. Applying the fixed effect Model (FEM) resulted in a statistically significant effect in favor of the control group (85 participants, MD -4.74 95% CI [− 8.30 to − 1.18], *p* = 0.002) with moderate heterogeneity (I^2^ = 51%). When a pre-specified random effects model (REM) was used, there was no difference between groups in pooled effects for fear of falling (85 participants, MD -4.01, 95% CI [− 10.4, 2.05], *p* = 0.51) with moderate heterogeneity (I^2^ = 51%). Using the GRADE approach, we assessed the quality of the evidence for this outcome to be very low due to a risk of performance bias (unblinded participants and personnel) and imprecision of results resulting from the very small number of participants.

**Risk of falling** The overall effect of PDHA on reducing risk of falling was not statistically significant (523 participants, RR 0.88, 95% CI [0.69 to 1.13], *p* = 0.32), I^2^ = 0%). Included were patients with mixed or unspecified diagnoses, respectively, hip fractures and stroke [1, 8, 23, 24, 53]. The quality of evidence was assessed as moderate because considerable harm and benefit were included in the confidence intervals of all the studies. We were therefore concerned with regard to imprecision.

**Risk of readmission** Pooling five studies showed no statistically significant effect on the reduction of readmissions throughout an average of 5 months after receiving PDHA (590 participants, RR 1.09, 95% CI [0.64 to 1.87], *p* = 0.70, I^2^ = 43%) in patients with unspecified or mixed diagnoses or stroke, respectively [7, 8, 23, 24, 53] . Applying the GRADE approach, the quality of evidence was assessed as moderate because significant harm and benefit were included in the confidence intervals of all the studies. For this reason, we were concerned with regard to imprecision.

### Outcomes from single studies

**Overall independence** was assessed with the Modified Ranking Scale [59] in one study with a missing significant difference between the groups at one month after discharge (16 participants, MD -0.20 95% CI [− 0.65 to 0.25], *p* = 0.38) [2].

### Psycho-social outcomes

One study reported on three different psycho-social outcomes, although all had missing significant differences at one month after discharge**: Emotional distress** in medical settings was measured through the GHQ-28 [42, 61] in 85 participants (in the intervention group with median 19; IQR 12.25–23.75 vs. median 23; IQR 15.5–31.5 in the control group; *p* = 0.10). **Depression** was measured through The Stroke Aphasic Questionnaire [43] in 85 participants (in the intervention group with median 6; IQR 3.25–9.75 vs. median 7; IQR 4–11 in the control group; *p* = 0.37). **Caregiver strain** was measured though the Caregiver Strain Index [44] in 85 participants (in the intervention group with median 5.5; IQR 1.75–7 vs. median 6; IQR 5–8 in the control group; *p* = 0.11).

### Process outcomes

The number of recommendations was reported in two studies with significant increases in the number of modifications in the intervention group compared to the control group at 30 or 90 days after discharge, respectively (average number of modifications 2.8 (1.6 to 3.9), *p* < .001 in one study and range 0–13 in intervention vs. 0–7 in controls, *p* = 0.001 in another study) [7].

### Admissions to hospitals and care facilities

The number of emergency department visits was reported in one study with missing significant differences between the groups at 90 days after discharge (337 participants; RR = 1.06, 95% CI [0.73 to 1.55], *p* = 0.73 [7].

One study (86 participants) reported missing significant differences in the number of institutionalizations after 12 months (60 participants, RR = 0.58; 95% CI 0.26 to 1.27; *p* = 0.17) [24].

The number of patients receiving community support was reported in one study, which stated that, three months after discharge, a total of three patients across groups received community support (seven patients across groups received support at baseline) [23].

### Qualitative synthesis

Based on four comprehensive descriptive themes, seven analytical themes were identified regarding the barriers and facilitators of the PDHA process. Details are reported in Additional file 7 (Summary of the descriptive themes) and Additional file 8 (Overview on analytical themes).

#### Barriers and facilitators in PDHA process, analytical themes

##### The safety assessment of the home environment

Participants highlighted the importance of safety after hospital discharge [30]. The aim was to identify any required provisions and adaptations before going home and to identify and eliminate risks within the home [3, 30, 31, 37], as well as to assess whether the home environment was suitable for the required equipment [37]. The facilitators were the structured identification of risk factors and patients'/family’s awareness of these factors following education, therefore enabling practical recommendations [30]. VR was identified as a useful tool to educate patients in order to identify and discuss risk factors, thus increasing patients’ awareness [3]. Therefore, we inferred the implication: 1 “Use environmental assessments together with patients to provide education about hazards.”

##### Functional assessment of the patient at home as a reality check

The aim of a functional assessment in general was to assess whether the patient is able to manage within his / her home [37]. On the whole, the predischarge home visit was a chance for therapists to gain a realistic view of the patients’ functions [30, 33–37]. But this also applied vice versa: “It’s making them [the patients] aware of that impact and how they might be able to overcome the problems they will encounter. [...] We do get patients who say ‘Oh, once I’m home I’ll be fine...’, but I don’t think they’ll always appreciate the limitations they’re going to encounter.” [37]. PDHA gave information on future therapy sessions and helped to tailor individual rehabilitation goals [33–35]. Visiting their home motivated patients to do the therapy so that they could return home [30, 31, 33, 35, 37]. Performance tests at home can cause the patients to become anxious about failure, so the social skills of OTs are definitely needed [31, 37]. However, the preparations for the functional requirements for carrying out activities of daily living at home in the context of PDHA offered the chance to reduce anxiety [30]. We inferred the implications: 2 Conduct a functional assessment that includes the living reality of the patient and helps the patient to find individual participation goals for therapy, and 2.1 Consider potential patient anxieties regarding the assessment situation.

##### Intervention planning and evaluation

Novice therapists in particular struggled with the aim and content of PDHA [30]. The actual timing of PDHA was highly dependent on organizational factors and resource availability [33, 34, 37]. There were often pragmatic aspects, like the availability of supportive network, patients’ preferences or “gut feeling” to consider when deciding about whether or not to conduct a PDHA [30, 34–36]. Working with community players often led to dissatisfaction with devices [35], without having the chance to follow up with the patient [34]. The facilitators were identified as: clear aims and assessment tasks, early patient identification and planning and a decision support tool [34], further use of standardized protocols during PDHA and collaboration with community services [30], as well as a formal evaluation after the PDHA [33]. The use of a digital interface to transmit environmental information could encourage the communication between the various stakeholders [9]. Therefore, we derived the implication 3: Use standardized procedures and materials to guide the PDHA process. Digital solutions might support the collaboration between hospital and community service providers.

##### Patient information about the home assessment procedure

Older people felt insufficiently informed prior to and after the home visit. Lack of information about the aims, the outcomes and the next steps of the process of PDHA made them feel insecure and anxious and excluded from the process [29, 31, 37]. Even during the home visit, there were situations in which the carer, but not the patient, was included in the process [31]. Written information about PDHA was seen as a facilitator by patients [29, 33]. Some patients and therapists felt a lack of real informed choice about the assessment [31]. Therefore we derived the implication 4. Provide adequate (verbal and written) patient information about aim, process, assessment, results and consequences of the predischarge home assessment.

##### Patients’ and family carers’ acceptance of home modifications and aids

The concerns of the patients that the OT’s modifications might hinder them in performing ADL in the usual and preferred way was identified as a barrier [31]. The use of a patient’s know-how on where to use an aid most effectively in their home environment was a facilitator for acceptance [35]. The lack of imagination regarding home modifications and adaptations [3] was seen as a barrier for acceptance. OTs and older people estimated that the use of visualization with a 3-D interior design software application would enable patients to better understand assistive technologies and adaptations [32]. OTs considered a virtual reality tool as superior to drawings and photographs [32]. Consequently, a more clear visualization was seen as a facilitator for OTs to communicate better about modifications [32]. In addition, a clear visualization as a joint basis for discussion was seen as a facilitator to include patients in the decisions about home modifications and aids, giving them a chance to give immediate feedback on proposed changes, thus leading to shared decision-making [3, 32]. Therefore we inferred implication 5: Provide tailored adaptations based on shared decision-making and involve explicitly patients’ ideas, solutions and expectations in planning home modifications, and 5.1 Provide appropriate visualization for discussing recommended aids and home modifications.

##### Matching PDHA and clinical patient conditions

Different patient conditions in terms of diagnosis and related kinds of impairments as well as the levels of impairment may be factors that facilitate or inhibit the performance of certain PDHA approaches. For example, sensory and visual limitations might be an indication for a home visit. However, the same limitations may have an adverse effect on the use of a virtual home assessment. Too low or too high levels of functional limitations spoke rather against home visits and in favor of ward-based assessments or access visits. Summing up, different patient conditions required different approaches for assessment. We inferred implication 6: Tailor the intervention components and mode of delivery to patients’ level and kind of impairments.

##### Context factors in daily routine of PDHA

Many of the qualitative studies identified factors that may have a beneficial or impeding effect on the decision of whether and how to conduct PDHA. Lack of resources (staff, time, secretarial backup, technical resources for virtual assessment) hampers the process of organization and execution [3, 29, 31, 34, 36, 37]. A virtual approach to PDHA could partially overcome some of the obstacles (e.g. out of hospital catchment zone, car availability, safety requirements for allowing a home visit with patient) [32]. Factors such as risks while making home visits and the organization of appropriate PDHA attendants have an impact on the process of PDHA [30, 34, 35, 37]. Therefore, we derived the implication 7: Consider specific context factors in PDHA-design.

### Integrative synthesis

An overview of the results of the analysis at the individual study level with regard to the respective qualitative results (whether the PDHA intervention had considered implications 1–7) and the outcome effects in the patient outcomes is shown in Table 3.

**Table 3:** Synthesis of practice implications and RCT interventions

Integrative synthesis of qualitative and quantitative results in studies in PDHA																				
			Implications for interventions									Outcomes								
Reference	Setting, diagnosis	Intervention	1	2	2.1	3	4	5	5.1	6	7	IADL/ADL, scale: NEADL	IADL/ADL, Various scales	Quality of Life, scale: EQ-5D	Quality of Life various scales	Mobility	Fear of Falling	Risk of falling	Risk of readmission	AE
												Effect (MD or RR), 95% CI								
**Studies**																				
[7]	Acute care, unspecified	Pre- and post- discharge visit + follow up phone calls	Nr	Un	Nr	Ad	Nr	Nr	Nr	Nr	Ad	MD-0.30 [− 1.32 to 0.72]	MD-0.06 [− 0.27 to 0.15]	–	–	–	–	**–**	RR 1.12 [0.76 to 1.66]	–
	Stroke rehabilitation unit, stroke	Predischarge home visit	Un	Ad	Nr	Nr	Nr	Un	Nr	Nr	Ad	MD −5.50 [−15.58 to 4.58]	MD −0.23 [− 0.66 to 0.20]	MD 0.03 [−9.40 to 9.46]	MD 0.00 [− 0.41 to 0.41**]**	–	–	RR 1.41 [0.67 to 2.98]	RR 3.91 [0.88 to 17.46]	–
[22, 25]	Acute care, hip fracture	Individual daily training including use of technical aids + predischarge home visit + instruction from physiotherapist how to walk with technical aids	Nr	Nr	Nr	N	Nr	Ad	Nr	Nr	Nr	–	MD 0.23 [− 0.19 to 0.66]	–	MD − 0.27 [− 0.70 to 0.16]	–	–	–	–	–
[23]	Rehabilitation unit, mixed	Predischarge home visit	Ad	Ad	Nr	Ad	Nr	Un	Nr	Nr	Nr	MD 13.40 [6.88 to 19.92]	MD 2.30 [0.51 to 4.10]	–	MD 1.50 [0.00 to 3.00]	MD 0.27 [− 0.98 to 1.51]	MD 7.00 [− 17.36 to 31.36]	RR 0.50 [0.06 to 3.91]	RR 0.33 [0.02 to 6.65]	–
[53]	Acute care and rehabilitation, hip fracture	Home assessment by occupational therapist prior to discharge	Un	Ad	Nr	Ad	Nr	Un	Nr	Ad	Nr	MD − 0.50 [− 3.47 to 2.47]	MD −0.08 [− 0.59 to 0.43]	MD 0.00 [− 0.13 to 0.13]	MD 0.00 [− 0.51 to 0.51]	–	MD − 1.20 [−6.72 to 4.32]	RR 0.83 [0.47 to 1.45]	RR 0.55 [0.25 to 1.18]	0
[1]	Geriatric acute care, unspecified	Predischarge home visit + post-discharge follow up visit(s) + comprehensive in-hospital geriatric assessment	Ad	Nr	Nr	Un	Nr	Un	Nr	Ad	Nr	–	–	–	–	–	–	RR 0.84 [0.63 to 1.12]	–	–
[24]	Geriatric acute care, unspecified	Predischarge home visit + contacting potential social support + physical therapy + therapeutic modifications	Ad	Nr	Nr	Nr	Nr	Un	Nr	Nr	Ad	–	MD −2.48 [− 3.16 to − 1.80]	–	–	–	–	RR 0.87 [0.50 to 1.49]	RR 1.50 [0.61 to 3.69]	–
[2]	Stroke ward / acute care, stroke	Predischarge Virtual Home Assessment	Ad	Nr	Nr	Un	Nr	Ad	Ad	Un	Ad	MD 12.00 [− 10.57 to 34.57]	MD 0.49 [− 0.51 to 1.49]	MD 0.19 [− 0.08 to 0.46]	MD 0.64 [− 0.37 to 1.65]	MD 2.24 [0.91 to 3.56]	MD − 7.80 [− 12.54 to − 3.06]	–	–	**–**

Implications: 1) Use environmental assessments together with patients to provide education about hazards. 2) Conduct a functional assessment that includes living reality of the patients and helps the patient to find individual participant goals for therapy. 2.1) Consider potential patient anxieties regarding the assessment situation. 3) Use standardized procedures and materials to guide the PDHA process. Digital solutions could support the collaboration between hospital and community service providers. 4) Provide adequate (verbal and written) patient information about aim, process, assessment, results and consequences of the predischarge home assessment. 5) Provide tailored adaptations based on shared decision-making and involve explicitly patient’s ideas, solutions and expectations in planning home modifications. 5.1) Provide appropriate visualization for discussing recommended aids and home modifications. 6) Tailor the intervention components and mode of delivery to patients’ level and kind of impairments. 7) Consider specific context factors in PDHA-design

*AE* Adverse effects of intervention, *Ad* Addressed, *Un* Unsure, *Nr* Not reported

## Discussion

This review investigated the impact of PDHA on functional outcomes associated with a successful return to community living for patients with various diagnoses. It also identified barriers and facilitators of the PDHA process from which recommendations for clinical practice could be derived.

### Improving patient outcomes with PDHA

Overall, there is a very low to moderate quality of evidence that PDHA might not result in any difference in patient outcomes when compared to usual care. There were only a few studies, and each of them investigated a variety of outcomes.

PDHA seems to have no impact on the quality of life. This result is in line with the systematic review by Lockwood et al. [4]. Although we included two additional RCTs and excluded one cohort study, our analysis also showed only a small overall effect size in favor of PDHA. However, the quality of the evidence for this is very low to low. Further studies with a robust sample size are required that are powered to assess effects in quality of life as a primary outcome.

**Mobility** To our knowledge, the evidence on the effect of PDHA on mobility was assessed for the first time in our review. Since there were only two studies with different outcome measures and very small sample sizes, the quality of evidence is very low about an effect of PDHA on mobility.

**Risk of falling** Since we only included randomized trials and excluded cohorts, our results do not confirm any effects of PDHA on risk reduction for falls in contrast to the findings of Lockwood [53]. The quality of the evidence for this finding is moderate. The few included studies reported conflicting results with large confidence intervals, so the body of evidence is still unclear. Therefore, further research is needed to confirm a possible effect.

**Fear of falling** To our knowledge, the evidence on the effect of PDHA on the fear of falling was assessed for the first time in our review. Our finding regarding the fear of falling is contrary to the evidence of an effect of PDHA on the reduction of falls, which Lockwood et al. had found. Our finding indicated that the fear of falling increases to a small extent. However, there is only low quality of evidence from two small studies showing a slight but significant increase of fear of falling in the intervention group when compared to usual care. This is in contrast to existing literature, which assumes that an increased fear of falling contributes to an increased risk of falling [62]. Our findings might be explained by the fact that an element of PDHA is to increase patients’ awareness of the potential risk of falling at home, which might also result in an increased fear of falling. This needs further investigation and should be considered when conducting the PDHA and the measures of discharge planning that result from the PDHA.

**IADL/ADL** When pooling various IADL measures and ADL measures of seven RCTs together, there was no effect of PDHA on IADL/ADL. Pooling only the studies that used the NEADL measure also resulted in no effect of PDHA. The quality of the evidence of our analysis is low although we added three additional RCTs, comparing to an earlier review by Lockwood et al. [4]. These authors suggested a benefit in ADL, also with a low quality of the evidence. These differences can be explained by the different approaches used: Lockwood et al. made a distinction between activity and participation measures. Since these constructs are very closely related at the level of measures for ADL, we decided not to differ between activity and participation measures. Since improving independence in everyday living is a core objective of PDHA, these results seem surprising. One reason might be the appropriateness of the chosen outcome measures used in RCTs [63]. The outcome measures included a range of items that are unlikely to be affected by PDHA interventions (e.g., items related to various activities outside the living environment or items for the assessment of communication functions or mental functions). A definition of desirable activity and participation items that are operationalized for each individual patient could make a measurement more sensitive and thus make changes more visible [63]. Standardized measures for patient goal attainment (e.g., The Canadian Occupational Performance Measure, COPM [58]) or single items from validated ADL scales would be conceivable here. At the same time, such measures would enhance patient involvement, which is believed to be fundamental to occupational therapy practice and the discharge planning process [64, 65].

Our findings on the effects of PDHA on risk of readmission for people who received a PDHA intervention did not show any difference compared to patients who received usual care. This result is in line with the existing literature [4]. Since we included an additional RCT with a large sample size, while excluding the cohort study, the quality of the evidence increased from “low” according to Lockwood et al. to “moderate”. A PDHA is expected to reduce risk of readmission to the hospital by preventing falls and their consequences. This was probably the reason why the majority of study authors chose this outcome measure for their RCTs. However, there are a number of other events causing readmission to hospital, which cannot be affected by PDHAs (e.g., relapse or aggravation of a previously known condition, complications and drug-related adverse events [66]). Therefore, risk of falling and fall-related consequences might be more appropriate outcome measures for assessing the effects of PDHA than readmission to hospital.

In this review, we identified potential factors for effectiveness from the views of stakeholders involved in the PDHA process; this method showed that some clear recommendations for practice could be developed systematically.

We included seven additional qualitative studies in the analysis of the barriers and facilitators of the PDHA process compared to earlier syntheses [4, 10]. Various implications were derived from the analysis of the qualitative studies as being meaningful criteria for PDHA design and implementation. These criteria - the necessity of considering the identification of hazards, the functional assessment in the context of the real home environment, and the inclusion of the patient’s participation goals and priorities in the assessment - are congruent with those criteria used in a previous study [67]. While this earlier review on environmental interventions defined these criteria from the best practice view of therapists, we were able to derive them systematically from the perspectives of therapists, patients and relatives. In addition, we were able to identify clinical factors influencing the execution of PDHA. The qualitative studies provide indications of the patient groups for which therapists consider a PDHA to be appropriate, especially in terms of level and type of impairment. From the quantitative studies, no firm conclusions can yet be drawn regarding the effect of PDHA on different patient groups. Further research is needed in this area. None of the included RCTs addressed all of the above mentioned meaningful aspects of intervention design. This illustrates that a modification of PDHA to improve patient-centeredness is indicated and might explain the missing effects on the investigated outcomes. PDHAs themselves fulfill all the characteristics of complex interventions, especially when a PDHA is part of the discharge management (e.g., involving a variety of stakeholders, organizational levels and outcomes) [6, 18]. Nevertheless, none of the included studies reported to have taken into account the current recommendations for the development of complex interventions [18]. Rather than the evaluation of existing PDHA approaches, an adaptation of PDHA interventions is needed, including a sound description of the context, the consequent inclusion of the user perspective as well as the current evidence.

In this review, qualitative studies focusing on general home modifications, regardless of the setting in which they were conducted, were excluded. However, as an integral part of a PDHA intervention, the stakeholders’ view of home modifications should also be examined in the future and the relevant implications for the design of PDHA should be derived.

#### Limitations

Our review has limitations owing to the shortcomings of the underlying studies. We decided to forgo subgroup analyses due to the small number of studies in this field. In order to take the heterogeneity of the studies into account a random effects model was used for the analysis. For the meta-analysis, we used the last time of follow-up, which differs for the various outcomes, partly considerably (from 1 month to 12 months). PDHAs could have different effects over this time period in the different outcomes. This was not accounted for in the meta-analysis and may have an impact on the results. The experiences and beliefs of the participants in the included qualitative studies were shaped by the context of specific healthcare and insurance systems and may not be valid in other regions. The findings of the integrative synthesis must be interpreted with caution. First, the implications derived from the individual studies by the thematic analysis have to be considered on the background of their particular study samples in their respective context and therefore they lack generalizability. We tried to overcome this limitation by including views from different stakeholder groups and different contexts of PDHA in a thematic analysis and distinguished between the perspectives of the different stakeholder groups. Second, in some studies it might not be possible to distinguish whether the implications were ultimately not taken into account during the implementation, or whether they were only insufficiently reported. Our comprehensive search strategy minimized the risk of missing studies, as we searched through the reference lists of systematic reviews, conducted a forward citation search and searched trial registers. A language bias due to the English and German language restriction cannot be ruled out. Further valuable strengths of our review include an unlimited search period and the screening and critical appraisal by two independent scientists.

## Conclusions

This systematic review revealed very low to moderate quality of evidence that PDHAs might have no impact on patient outcomes that are associated with a successful return to community living. Therefore, no conclusion can be drawn as to whether PDHA should be performed or to what kind of PDHA is required. Furthermore, additional research is needed to assess the effectiveness of PDHA on different patient populations as they may respond differently on the intervention. However, implications in intervention design can be drawn from qualitative studies of stakeholders’ perspectives on facilitators and barriers in the process of PDHA. Current RCTs partially consider these implications for complex PDHA interventions. For future PDHA, careful intervention development should be based on the existing qualitative evidence of stakeholder views. In future research, sufficiently robust RCTs using valid effect size estimates are needed in order to assess the effects of PDHAs. The use of appropriate outcome measures, reflecting the users’ demands on the PDHA process as well as the individual character of the patients’ adjustment to function and home environmental requirements, might improve the evaluation of the interventions’ effectiveness. Future studies should describe the PDHA intervention adequately, including how it is embedded in the discharge management to improve the dependability and to contribute to a better understanding of how the intervention might work.

## Supplementary Information

**Additional file 1.** Search strategy and excluded references. Search terms and search strategy exemplary for MEDLINE via PubMed.

**Additional file 2.** Outcome hierarchy for ADL IADL measures. Authors’ pre-specified hierarchy of ADL and IADL measures for meta-analysis.

**Additional file 3.** Detailed ROB single studies. Risk of bias assessment on single study level, detailed explanation per outcome, additional graphics.

**Additional file 4.** Quality appraisal qualitative studies. Quality appraisal on single study level in qualitative studies.

**Additional file 5.** GRADE pooled effects. Detailed explanation of GRADE judgment per outcome.

**Additional file 6.** Forest plots. Graphical display of meta-analysis with effects in all pooled outcomes.

**Additional file 7.** Descriptive themes. Summary of findings in qualitative studies on level of descriptive themes.

**Additional file 8.** Analytical themes. Summary of analytical themes from qualitative synthesis with related barriers and facilitators and derived implications.

## Data Availability

The data published may be found in the original manuscripts cited in the list of references. The data extraction sheets from the RCTs and the qualitative studies are available from the corresponding author on reasonable request.

## Abbreviations

3-D: Three-dimensional
ADL/IADL: Activities of Daily Living/Instrumental Activities of Daily Living
COPM: Canadian Occupational Performance Measure
EQ-5D: Measure of health status from the EuroQol Group (EQ-5D)
FE: Fixed Effect Model
FES-I: Falls Efficacy Scale-International
FU: Follow-up
GRADE: The Grading of Recommendations Assessment, Development and Evaluation working group
MRS: Modified Ranking Scale
n.a.: Not applicable
n.r.: Not reported
NEADL: Extended Activities of Daily Living Scale
OT: Occupational Therapy
PDHA: Predischarge home assessment
RCT: Randomized Controlled Trial
RE: Random Effects Model
RMI: The Rivermead Mobility Index
SMAF: The Functional Autonomy Measurement System
SWED-QUAL: The Swedish Health-Related Quality of Life Survey
VR: Virtual Reality
vs: Versus
