# Supplementary material for: Effects, barriers and facilitators in predischarge home assessments to improve the transition of care from the inpatient care to home in adult patients: an integrative review
Source: BMC Health Serv Res. 2021 Jun 2;21:540. doi: 10.1186/s12913-021-06386-4 (PMC8170965; doi:10.1186/s12913-021-06386-4)
Supplement: Supplementary file 1 — Additional file 1. Search strategy and excluded references. Search terms and search strategy exemplary for MEDLINE via PubMed. [file 12913_2021_6386_MOESM1_ESM.docx]

| **search strategy exemplary for MEDLINE via PubMed** | | |
| --- | --- | --- |
|  |  | discharge |
|  | ***OR*** | inpatient |
|  | ***OR*** | "subacute care" |
|  | ***OR*** | "acute care" |
|  | ***OR*** | rehabilitation [MeSH Term] |
|  | | |
| ***AND*** |  | "house calls"[Mesh] |
|  | ***OR*** | "home visiting" |
| ***OR*** | "home visit" |  |
| ***OR*** | "environmental assessment" |  |
| ***OR*** | "assessment visit" |  |
|  | ***OR*** | "home safety" |
|  | ***OR*** | "home modification" |
|  | ***OR*** | "environmental modification" |
|  | ***OR*** | "weekend passes" |
|  | ***OR*** | "weekend pass" |
|  | | |
| *(discharge OR inpatient OR "subacute care" OR "acute care" OR rehabilitation [MeSH Term])AND("House Calls"[Mesh] OR "home visiting" OR "home visit" OR "environmental assessment" OR "assessment visit" OR "home safety" OR "home modification" OR "environmental modification" OR "weekend passes" OR “weekend pass”)* | | |
| ***Results:*** | | [983](https://www.ncbi.nlm.nih.gov/pubmed/?cmd=HistorySearch&querykey=14) (*21.06.2018 – 16:00)* |

**Additional file 1**

***Search strategy and characteristics of excluded references***

|  | **Search Strategy** |
| --- | --- |
| **1** | discharge |
| **2** | inpatient |
| **3** | "subacute care" |
| **4** | "acute care" |
| **5** | rehabilitation [MeSH Term] |
| **6** | **1 OR 2 OR 3 OR 4 OR 5** |
| **7** | "house calls"[Mesh] |
| **8** | "home visiting" |
| **9** | "home visit" |
| **10** | "environmental assessment" |
| **11** | "assessment visit" |
| **12** | "home safety" |
| **13** | "home modification" |
| **14** | "environmental modification" |
| **15** | "weekend passes" |
| **16** | "weekend pass" |
| **17** | **7 OR 8 OR 9 OR 10 OR 11 OR 12 OR 13 OR 14 OR 15 OR 16** |
| **18** | **6 AND 17** |

| **Excluded references** | |
| --- | --- |
| Aplin, 2013 | study explores stakeholders’ views on home modifications in general, not in the scope of predischarge home assessments |
| Barras, 2005 | study design |
| Boonstra, 2005 | study design |
| Cahill, 2008 | not found by full text search |
| Cameron, 2010 | type of publication |
| Chibnall, 2011 | study design |
| Clarke, 1995 | study design |
| Cumming, 1999 | time point of home visit not only pre-discharge |
| Di Monaco, 2008 | home visit afetr discharge |
| Durham, 1993 | study design |
| Fellows , 2011 | type of publication |
| Gursen, 2003 | time point of home visit unclear |
| Gustafsson, 2011 | type of publication |
| Hamrick, 2013 | type of publication |
| Hibbert, 2008 | type of publication |
| Johnston, 2010 | study design |
| Laver, 2013 | type of publication |
| Lau, 2018 | study explores stakeholders’ views on home modifications in general, not in the scope of predischarge home assessments |
| Linkewich, 2010 | type of publication |
| Lo Bianco, 2020 | study explores stakeholders’ views on home modifications in general, not in the scope of predischarge home assessments |
| Lockwood, 2015 | study design |
| Mason, 1985 | study design |
| McCluskey, 2008 | type of publication |
| McDonald, 2003 | type of publication |
| McKale, | study design |
| Money, 2015 | The aim of this study was to explore community  dwelling older adults’ perceptions of using a 3D-software in  terms of its potential usefulness, ease of use, and actual  use and to consider the potential barriers and opportunities  of using the software application as an assistive tool  within the pre-discharge home visits process. However, the participants were healthy adults with no actual experience or link to PDHA-Process. |
| Mountain, 2002 | study design |
| Napolitan, 1999 | not found by full text search |
| Nikolaus, 1995 | study design |
| Nix, 2017 | study design |
| Olin, 1982 | type of publication |
| Patterson, 1999 | study design |
| Penrose, 1988 | type of publication |
| Rogers, 1989 | type of publication |
| Renforth, 2004 | study design |
| Rosenblatt, 1986 | study design |
| Sanford, 2004 | Wrong study question |
| Sim, 2015 | study design |
| Smith Toby, 2015 | study design |
| Swanton, 2017 | type of publication |
| Threapleton, 2016 | type of publication |
| Walker, 2002 | type of publication |
| Whitaker, 1986 | study design |
| Wang, 2017 | study design |
| Welch, 2005 | study design |
| Wilson | study design |
| Wiwanitkit, 2015 | type of publication |
| Yeung, 2012 | not found by full text search |
| Wallace, 1988 | type of publication |
