# Supplementary material for: Effects, barriers and facilitators in predischarge home assessments to improve the transition of care from the inpatient care to home in adult patients: an integrative review
Source: BMC Health Serv Res. 2021 Jun 2;21:540. doi: 10.1186/s12913-021-06386-4 (PMC8170965; doi:10.1186/s12913-021-06386-4)
Supplement: Supplementary file 2 — Additional file 2. Outcome hierarchy for ADL IADL measures. Authors’ pre-specified hierarchy of ADL and IADL measures for meta-analysis. [file 12913_2021_6386_MOESM2_ESM.docx]

# Additional file 2

# Pre-specified hierarchy of outcome measures for ADL and IADL

The constructs activities and participation are difficult to separate. Therefore the research group tried to prioritize instruments, which represent the best middle way and are not too much emphasized on simple ADL functions (e.g. bladder control) or too complex (e.g. travel out of town). The research group discussed the various measures with respect to the items they contain and considered the NEADL as the most appropriate outcome measure. The order of relevance of the other measures was discussed and agreed by consensus.

1. NEADL [1]
2. IADL [2]
3. FIM 5.1 [3]
4. SMAF [4]
5. RNLI [6]
6. Barthel Index [5]
7. Activities of Daily Living Scale (Klein-Bell) [7]
8. Activities of Daily Living Scale (Katz) [8]

References

1. Nouri, F., Lincoln, N.: An extended activities of daily living scale for stroke patients. Clinical rehabilitation(1), 301–305 (1987)

2. Lawton, M.P., Brody, E.M.: Assessment of older people: Self-maintaining and instrumental activities of daily living. gerontologist **9**, 179–186 (1969)

3. Linacre, J.M., Heinemann, A.W., Wright, B.D., Granger, C.V., Mamilton, B.B.: The Structure and Stability of the Functional Independence Measure. Archives of Physical Medicine and Rehabilitation(75) (1994)

4. Hebert, R., Guilbault, J., Desrosiers, J., Debuc, N.: THE FUNCTIONAL AUTONOMY MEASUREMENT SYSTEM (SMAF): A CLINICAL-BASED INSTRUMENT FOR MEASURING DISABILITIES AND HANDICAPS IN OLDER PEOPLE. Geriatrics Today: Journal of Canadian Geriatric Society(September), 1–7 (2001)

5. Collin, C., Wade, D.T., Davies, S., Horne, V.: The Barthel ADL Index: A reliability study. International Disability Studies **10**(2), 61–63 (1988). doi: 10.3109/09638288809164103

6. Tooth, L.R., McKenna, K.T., Smith, M., O'Rourke, P.K.: Reliability of scores between stroke patients and significant others on the Reintegration to Normal Living (RNL) Index. Disability and rehabilitation **25**(9), 433–440 (2003). doi: 10.1080/0963828031000069726

7. Klein, R.M., Bell, B.: Self-care skills: behavioral measurement with Klein-Bell ADL scale. Archives of Physical Medicine and Rehabilitation **63**(7), 335–338 (1982)

8. Katz, S., Downs, T.D., Cash, H.R., Grotz, R.C.: Progress in development of the index of ADL. The Gerontologist **10**(1), 20–30 (1970). doi: 10.1093/geront/10.1_part_1.20
