# Supplementary material for: Effects, barriers and facilitators in predischarge home assessments to improve the transition of care from the inpatient care to home in adult patients: an integrative review
Source: BMC Health Serv Res. 2021 Jun 2;21:540. doi: 10.1186/s12913-021-06386-4 (PMC8170965; doi:10.1186/s12913-021-06386-4)
Supplement: Supplementary file 3 — Additional file 3. Detailed ROB single studies. Risk of bias assessment on single study level, detailed explanation per outcome, additional graphics. [file 12913_2021_6386_MOESM3_ESM.docx]

**Additional file 3**

***Details on risk of bias assessment of the single studies***

**Judgement on present studies**

| **Clemson, 2016: Occupational Therapy Pre-discharge Home Visits in Acute Hospital Care: A Randomized Trial** | | |
| --- | --- | --- |
| **Random sequence generation?** | **Low risk** | - Randomization was appropriate using a computer generated random block sequence. - The schedule was developed by a non-involved researcher. |
| **Allocation concealment?** | **Low risk** | - Allocation sequence concealed using a password-protected website - Maintained by an independent service |
| **Blinding of participants and personnel?** | **Unclear risk in IADL/ADL** | - No sufficient information for judgement. |
|  | **Low in risk of readmission** | - No sufficient information for judgement, but clinical outcome. |
| **Blinding of outcome assessment?** | **Low risk** | - Masked researchers made follow-up telephone calls. - A research assistant conducted a blinded content analysis. |
| **Incomplete outcome data?** | **Low risk** | - All drop-outs and their reasons are reported. - The estimated sample size was not reached. - Intention-to-treat was used for analyses. All data are stated. |
| **Selective reporting?** | **Low risk** | - All pre-specified outcomes are reported. |
| **Other bias?** | **Unclear risk** | - Performing OTs significantly different (juniors /seniors) between groups. - 4 participants got home visits outpatient from non-study OTs after discharge |

| **Drummond, 2012: Occupational therapy pre-discharge home visits for patients with a stroke (HOVIS): results of a feasibility randomized controlled trial** | | |
| --- | --- | --- |
| **Random sequence generation?** | **Low risk** | - Randomization was appropriate using a web-based randomization program. |
| **Allocation concealment?** | **Low risk** | - Web based randomization-allocation not predictable. |
| **Blinding of participants and personnel?** | **Unclear risk in QoL** | - Insufficient information for judgement. |
|  | **Unclear risk in IADL/ADL** | - Insufficient information for judgement. |
|  | **Low risk in risk of fall/readmission** | - Insufficient information for judgement, but clinical outcome. |
| **Blinding of outcome assessment?** | **Low risk** | - The majority of data was entered and analyzed blind to group allocation. Only adverse events were reviewed reasonably unblinded. |
| **Incomplete outcome data?** | **Low risk** | - All drop-outs and their reasons are reported. - Intention-to-treat was used for analyses. All data are stated. - If more than 10% of the data were missing, the measure was coded as missing. If less than 10% were missing, the mean values were imputed. |
| **Selective reporting?** | **Low risk** | - All pre-specified outcomes are reported. |
| **Other bias?** | **Low risk** | - Not detectable. |

| **Hagsten, 2004/2006: Early individualized postoperative occupational therapy training in 100 patients improves ADL after hip fracture** | | |
| --- | --- | --- |
| **Random sequence generation?** | **Low risk** | - 50/50 opaque envelopes were sealed, numbered from 1 to 100, mixed by an external person and drawn consecutively |
| **Allocation concealment?** | **Low risk** | - Used opaque and sealed envelopes - 10 participants were excluded before the first assessment. For those 10, corresponding assignment cards were put in new envelopes, sealed and inserted in a blind manner among the remaining ones. Thus, the allocation could be concealed. |
| **Blinding of participants and personnel?** | **High risk**  **For QoL** | - It was not possible to hide the allocation from the nursing staff etc. |
|  | **High risk**  **For IADL/ADL** | - It was not possible to hide the allocation from the nursing staff etc. |
| **Blinding of outcome assessment?** | **Unclear risk** | - **Insufficient information** |
| **Incomplete outcome data?** | **High risk** | - >20% loss to follow-up. - Intention-to-treat analysis and other preventive steps not reported. |
| **Selective reporting?** | **Unclear risk** | - Outcomes insufficiently reported. |
| **Other bias?** | **Low risk** | • Not detectable. |

| **Lannin, 2007: Feasibility and results of a randomised pilot-study of pre-discharge occupational therapy home visits** | | |
| --- | --- | --- |
| **Random sequence generation?** | **Low risk** | - Randomization was appropriate using a computer generated allocation schedule. It was concealed in opaque envelopes. |
| **Allocation concealment?** | **Low risk** | - Consecutively-numbered envelopes by a non- involved person were used. - Baseline outcomes were assessed prior to randomization. - There were no significant differences at baseline although acute hospital length of stay prior to rehabilitation was longer in the control group. |
| **Blinding of participants and personnel?** | **Low risk in mobility** | - Unblinded participants, performance test was used for assessment of mobility. |
|  | **High risk in IADL/ADL** | - Unblinded participants, patient-reported outcome measure. |
|  | **High risk in fear of falling** | - Unblinded participants, patient-reported outcome measure. |
|  | **High risk for QoL** | - Unblinded participants, patient-reported outcome measure. |
|  | **Low risk in risk of readmission/fall** | - Unblinded participants, clinical outcomes |
| **Blinding of outcome assessment?** | **Low risk** | - The assessor was blinded. The data were coded to ensure confidentiality and blinding to group allocation. |
| **Incomplete outcome data?** | **Low risk** | - All drop-outs and their reasons are reported. - Intention-to-treat was used for analyses. - All data are stated. Missing data were omitted. |
| **Selective reporting?** | **Low risk** | - All pre-specified outcomes are reported. |
| **Other bias?** | **Low risk** | - Not detectable. |

| **Nikolaus and Bach, 2003: Preventing falls in community-dwelling frail older people using a Home Intervention Team (HIT): Results from the randomized Falls-HIT Trial** | | |
| --- | --- | --- |
| **Random sequence generation?** | **Low risk** | - Randomization was appropriate using random number sequence. |
| **Allocation concealment?** | **Low risk** | - Sealed envelopes containing group assignments were used. - The intervention and control groups did not differ clearly at baseline. |
| **Blinding of participants and personnel?** | **Low risk in risk of fall** | - Insufficient information for judgement, but clinical outcome. |
| **Blinding of outcome assessment?** | **Low risk** | - Interviewer was blinded to group allocation. But we are unsure if blinding is likely to have been maintained during the continuous phone follow-up. |
| **Incomplete outcome data?** | **Low risk** | - All drop-outs and their reasons are reported. Although there is a high rate of drop-outs, the estimated sample size was reached during follow-up. Intention-to-treat was used for all analyses. |
| **Selective reporting?** | **Low risk** | - All pre-specified outcomes are reported. - Although the study was not designed to examine fall-related injuries as an endpoint, the data was reported. |
| **Other bias?** | **Low risk** | - Not detectable. |

| **Pardessus, 2002: Benefits of Home Visits for Falls and Autonomy in the Elderly.** A Randomized Trial Study | | |
| --- | --- | --- |
| **Random sequence generation?** | **Low risk** | - Randomization was appropriate using random number tables. |
| **Allocation concealment?** | **Unclear risk** | - Intervention and control groups did not differ in baseline-assessment. - There is no information about allocation reported. |
| **Blinding of participants and personnel?** | **Unclear risk in IADL/ADL** | - Insufficient information for judgement. |
|  | **Low risk for risk of fall/readmission** | - Insufficient information, but clinical outcome. |
| **Blinding of outcome assessment?** | **Unclear risk** | - Insufficient information. |
| **Incomplete outcome data?** | **Low risk** | - Drop-outs are reported appropriate. - All outcomes are reported in text or tables. - The type of analysis is not clearly stated (intention-to-treat could be suggested). |
| **Selective reporting?** | **Low risk** | - All pre-specified outcomes are reported. |
| **Other bias?** | **Low risk** | - Not detectable. |

| **Threapleton, 2018: Virtually home: Feasibility study and pilot randomised controlled trial of a virtual reality intervention to support patient discharge after stroke** | | |
| --- | --- | --- |
| **Random sequence generation?** | **Low risk** | - Randomization was appropriate when using a web-generated list by an independent research administrator |
| **Allocation concealment?** | **Low risk** | - Group allocation using sealed opaque envelopes was revealed after completion of baseline assessment. - There were no significant differences between the groups at baseline. |
| **Blinding of participants and personnel?** | **Unclear risk in IADL/ADL** | - Insufficient information for judgement. |
|  | **Unclear risk in QoL** | - Insufficient information for judgement. |
|  | **Unclear risk in mobility** | - Insufficient information, patient rated outcome measure used. |
|  | **Unclear risk in fear of falling** | - Insufficient information, patient rated outcome measure used |
| **Blinding of outcome assessment?** | **Low risk** | - Blinded assessor did follow-up. |
| **Incomplete outcome data?** | **Low risk** | - Drop-outs are reported appropriately. >20% (2 out of 8) loss of follow-up equally in each group. Baselines were equal. |
| **Selective reporting?** | **Low risk** | - All pre-specified outcomes are reported. |
| **Other bias?** | **Low risk** | - 2 patients (25%) from control group also received a (non-virtual) home visit. Control intervention was contaminated. |

| **Lockwood, 2019/2020 Predischarge home visits after hip fracture: a randomized controlled trial/ Home visits by occupational therapists improve adherence to recommendations: Process evaluation of a randomised controlled trial** | | |
| --- | --- | --- |
| **Random sequence generation?** | **Low risk** | - Permuted blocks of four and six generated electronically |
| **Allocation concealment?** | **Low risk** | - concealed in sequentially numbered, sealed opaque envelopes prepared by a member of the research team not involved in recruitment or data collection. |
| **Blinding of participants and personnel?** | **Unclear risk in IADL/ADL** | - Insufficient information for judgement. |
|  | **Unclear risk in QoL** | - Insufficient information for judgement. |
|  | **Unclear risk in fear of falling** | - Insufficient information for judgement. |
|  | **Low risk for risk of fall/readmission** | - Insufficient information, but clinical outcome. |
| **Blinding of outcome assessment?** | **Low risk** | - Blinded assessor did follow-up. |
| **Incomplete outcome data?** | **Low risk** | - Drop-outs are reported appropriately. 12.5% loss of follow-up equally in each group. Baselines were equal. |
| **Selective reporting?** | **Low risk** | - All pre-specified outcomes are reported. |
| **Other bias?** | **Low risk** | - Not detectable. |
