# Supplementary material for: Effects, barriers and facilitators in predischarge home assessments to improve the transition of care from the inpatient care to home in adult patients: an integrative review
Source: BMC Health Serv Res. 2021 Jun 2;21:540. doi: 10.1186/s12913-021-06386-4 (PMC8170965; doi:10.1186/s12913-021-06386-4)
Supplement: Supplementary file 4 — Additional file 4. Quality appraisal qualitative studies. Quality appraisal on single study level in qualitative studies. [file 12913_2021_6386_MOESM4_ESM.docx]

**Additional file 4**

***Quality appraisal of qualitative studies***

| criterion/study | | Atwal, 2008 | Atwal, 2014a | Atwal, 2014b | Cameron, 2014 | Davis, 2019 | Godfrey, 2019 | Hibbert, 2008 | Nygard, 2014 | Threapleton, 2016 | Whitehead, 2014 |
| --- | --- | --- | --- | --- | --- | --- | --- | --- | --- | --- | --- |
| 1.1 | Research goal reported? | Yes | Yes | Yes | Yes | Yes | Yes | Yes | Yes | Yes | Yes |
| 1.2 | Relevance of research goal stated | Yes | Yes | Yes | Yes | Yes | Yes | Yes | Yes | Yes | Yes |
| 2. | Underlying qualitative paradigm | Interpretative approach | Not stated | Not stated | Not stated | No paradigm | No paradigm | No paradigm, thematic analysis | No paradigm | Not stated, thematic analysis | Not stated |
| 3. | Study design | Qualitative cross sectional | Qualitative cross sectional | Qualitative cross sectional | Qualitative longitudinal | Qualitative cross sectional | Qualitative cross sectional | Qualitative cross sectional | Qualitative longitudinal | Qualitative cross sectional | Qualitative cross sectional |
| 4. | Qualitative design appropriate | Yes | Yes | Yes | Yes | Yes | Yes | Yes | Yes | Yes | Yes |
| 5. | Qualitative method | Semi structured interview | Semi structured interview/ think aloud technique | Semi structured interview | In depth and semi structured interview/ focus groups | survey design with Semi structured questionnaire | In depth and semi structured interview/ focus groups | Semi structured interview | Interviews/ focus groups/ participant observation | Semi structured interview | Semi structured interview |
| 6.1 | Methods explicitly described | Yes | Yes | Yes | Yes | Yes | Yes | Yes | Yes | Yes | Yes |
| 6.2 | Modified methods during the study justified and described | methods not modified | methods not modified | methods not modified | methods not modified | methods not modified | methods not modified | methods not modified | methods not modified | methods not modified | methods not modified |
| 6.3 | Data collecting described | Yes | Yes | Yes | Yes | Yes | Yes | Yes | Yes | Yes | Yes |
| 6.4 | Saturation of data discussed | Yes | Yes | Yes | No | Yes | Yes | Yes | Yes | Yes | No |
| 7.1 | Selection of participants appropriate | Yes | Unclear | Yes | Unclear | Yes | Yes | Yes | Yes | Yes | Yes |
| 7.2 | Recruitment part of discussion | Yes | Yes | Yes | No | Yes | Yes | Yes | Yes | Yes | Yes |
| 8. | Researcher's influence on study discussed | No | No | Yes | No | No | Yes | Yes | Yes | No | Yes |
| 9. | Ethical issues adequately addressed | Yes | Yes | No | No | Yes | Yes | No | Yes | Yes | Yes |
| 10 | Rigorous data analysis | | | | | | | | | | |
| 10.1 | Analysis process described in detail | Yes | Yes | Yes | No | Yes | Yes | No | Yes | Yes | Yes |
| 10.2 | Major themes clearly presented in findings | Yes | Yes | Yes | Yes | Yes | Yes | Yes | Yes | Yes | Yes |
| 10.3 | Derivation of thematic analysis clearly stated | Yes | Yes | Yes | No | No | No | No | No | No | No |
| 10.4 | Explanation of data selection from original sample | Yes | Yes | Yes | Yes | No | Yes | No | Yes | Yes | Yes |
| 10.5 | Proper quotations from participants used | Yes | Yes | Yes | Yes | No | Yes | Yes | No | Yes | Yes |
| 10.6 | Description of diverse cases an minor themes | Yes | Yes | No | No | No | Yes | Yes | No | No | Yes |
| 11.1 | Adequate discussion of evidence | Yes | Yes | Yes | Yes | Yes | Yes | Yes | Yes | Yes | Yes |
| 11.2 | credibility of findings discussed | Yes | Yes | Yes | Yes | Yes | Yes | Yes | Yes | Yes | Yes |
| 11.3 | Findings discussed in relation to research question | Yes | Yes | Yes | Yes | Yes | Yes | Yes | Yes | Yes | Yes |
| 12. | Research is valuable | Yes | Yes | Yes | Yes | Yes | Yes | Yes | Yes | Yes | Yes |
