# Supplementary material for: Effects, barriers and facilitators in predischarge home assessments to improve the transition of care from the inpatient care to home in adult patients: an integrative review
Source: BMC Health Serv Res. 2021 Jun 2;21:540. doi: 10.1186/s12913-021-06386-4 (PMC8170965; doi:10.1186/s12913-021-06386-4)
Supplement: Supplementary file 5 — Additional file 5. GRADE pooled effects. Detailed explanation of GRADE judgment per outcome. [file 12913_2021_6386_MOESM5_ESM.docx]

**Additional File 5**

| **Judging the Quality of Evidence according to the GRADE Handbook [10]** | | |
| --- | --- | --- |
| **Grade domain** | **Judgment** | **Concerns about certainty domains** |
| ***Outcome: IADL/ADL various scales*** | | |
| Limitations in the design and implementation of available studies suggesting high likelihood of bias** | Seven studies [10, 16, 19, 20, 29, 34, 43, 53] referred to the outcome IADL/ADL.  All the studies had appropriate sequence generation. In one study[43], allocation concealment was unclear, which was not judged as a serious limitation. Blinding of participants and personnel were not sufficiently reported, which was judged as an unclear risk of bias in four studies. Blinding was not possible due to the nature of the intervention in two of the six studies and was rated as high risk. Biased rating of the measures was possible (rating scales), though not likely. Blinding of outcome assessors was unclear in two studies[19, 20, 43]. Considered as borderline serious. In one study the dropout [19, 20] rate was higher than 20%, and intention-to-treat analysis or preventive steps were not reported. This is considered borderline serious. Overall, the authors judged concerns as serious because of two possible sources of bias. | Serious, downgrade one level |
| Inconsistency of results | The direction and magnitude of the effect in IADL/ADL across the trials were conflicting. Overall, there were smaller effects in both directions. Two studies [29, 43] showed large effects, although in different directions, and one of these studies had low statistical power[29]. There was a large variability in point estimates and in the overlap in CIs and a large statistical heterogeneity (I^2^=90%). | Serious, downgrade one level |
| Indirectness of evidence | The study population across the studies varied in diagnoses and age. The mean age of the patients across all six studies was approximately 80 years, but the studies that reported ranges in age also reported on a wide range (34-95 years). The research question was for any diagnosis and adults ≥18 years. Therefore, outcome evidence from the included studies may be applicable on the population under investigation. The intervention of one study [53] (virtual predischarge home assessment with additional access visit, if required) differed somewhat from the other studies (predischarge-home assessment visit) and might differ from current practice. However, the intense use of technological advancement in occupational therapy is expected. No systematic differences in different countries or for different clinical settings could be detected. Therefore, we had no serious concerns about the directness of evidence with respect to the interventions. Outcome measures are judged as highly patient-important and were therefore seen as direct. The comparator in all of the studies was usual care, but usual care was not always described in detail and differs in different countries. This may possibly affect the certainty. This was judged as borderline serious.  Overall, there are no serious concerns about indirectness. | Borderline serious, do not downgrade |
| Imprecision | The total number of study participants for IADL/ADL was 655, which lies above the threshold regarding the optimal information size (IOS). However, appreciable harm and benefit were included, with wide confidence intervals. Therefore, we had serious concerns regarding imprecision. | Serious, downgrade one level |
| Likelihood of publication bias | Our literature search was comprehensive, there were no unpublished studies in the registries, and the studies reported both positive and negative outcomes. Therefore, we had no serious concerns. | Not serious |
| Overall judgment |  | ⨁OOO  Very low |
| ***Outcome: IADL/ADL NEADL*** |  |  |
| Limitations in the design and implementation of available studies suggesting high likelihood of bias** | Five studies [10, 17, 31, 37, 57] referred to the outcome IADL/ADL, measured by NEADL[41]. All the studies had appropriate sequence generation. In all of the studies, blinding of participants and personnel was hardly possible due to the nature of the intervention.Therefore, ROB was judged as unclear in5ive studies. ROB in two studies was high. Biased rating of the measures was possible (rating scales), though not likely. Considered as borderline serious. Not judged as a serious limitation. | Serious, downgrade one level |
| Inconsistency of results | Heterogeneity may be high (I^2^=79%). | Serious, downgrade one level |
| Indirectness of evidence | The study population across studies varied in diagnoses and age. The mean age of the patients across all three studies was approximately 80 years, but the studies that reported ranges in age also reported on a wide range (34-95 years). The research question was for any diagnosis and adults ≥18 years. Therefore, outcome evidence from the included studies may be applicable on the population under investigation. The intervention of one study [53] (virtual predischarge home assessment with additional access visit, if required) differed somewhat from the other studies (predischarge home assessment visit) and might differ from current practice. However, the intense use of technological advancement in occupational therapy is expected.  No systematic differences in different countries or for different clinical settings could be detected. Therefore, we had no serious concerns about the directness of evidence with respect to the interventions. Outcome measures are judged as highly patient-important and were therefore seen as direct. The comparator in all of the studies was usual care, but usual care was not always described in detail and differs in different countries. This may possibly affect the certainty. This was judged as borderline serious.  Overall, there are no serious concerns about indirectness. | Borderline serious, do not downgrade |
| Imprecision | The total number of study participants for IADL/ADL was 510, which lies above the threshold regarding the optimal information size (OIS).  Appreciable harm and benefit were included, with wide confidence intervals. We had serious concerns regarding imprecision. | Serious, downgrade one level |
| Likelihood of publication bias | Our literature search was comprehensive, there were no unpublished studies in the registries, and the studies reported both positive and negative outcomes. Therefore, we had no serious concerns. | Not serious |
| Overall judgment |  | ⨁OOO Very low |
| ***Outcome: Quality of Life EQ-5D*** | | |
| Limitations in the design and implementation of available studies suggesting high likelihood of bias** | Three studies [16, 34, 53] reported on EQ-5D quality of life.  In all studies, blinding of personnel and participants was not possible due to the nature of the intervention and was therefore judged as an unclear risk. Biased rating of the measures was possible (rating scales), though not likely. Considered as borderline serious. | Borderline serious, do not downgrade |
| Inconsistency of results | Statistical heterogeneity was low (I^2^=0%). We judged inconsistency as not serious. | Not serious |
| Indirectness of evidence | The study population across studies varied in diagnoses and age. The mean age of the patients across all three studies was approximately 80 years, but the studies that reported ranges in age also reported on a wide range (34-95 years). The research question was for any diagnosis and adults ≥18 years. Therefore, outcome evidence from the included studies may be applicable on the population under investigation.  The intervention of one study [53] (virtual predischarge home assessment with additional access visit, if required) differed somewhat from the other studies (predischarge-home assessment visit) and might differ from current practice. However, the intense use of technological advancement in occupational therapy is expected. No systematic differences in different countries or for different clinical settings could be detected. Therefore, we had no serious concerns about the directness of evidence with respect to the interventions. Outcome measures are judged as highly patient-important and were therefore seen as direct. The comparator in all of the studies was usual care, but usual care was not always described in detail and differs in different countries. This may possibly affect the certainty. This was judged as borderline serious.  Overall, there are no serious concerns about indirectness. | Borderline serious, do not downgrade |
| Imprecision | The total number of participants of 186 is less than the required OIS of 400. Appreciable harm and benefit were included in wide confidence intervals. We had serious concerns regarding imprecision. | Serious, downgrade two levels |
| Likelihood of publication bias | Our literature search was comprehensive, there were no unpublished studies in the registries, and the studies reported both positive and negative outcomes. Therefore, we had no serious concerns. | Not serious |
| Overall judgment |  | ⨁⨁OO  Low |
| ***Outcome: Quality of Life various scales*** | | |
| Limitations in the design and implementation of available studies suggesting high likelihood of bias** | Five studies [16, 20, 29, 34, 53] reported on quality of life with various scales.  In all of the studies, blinding of personnel and participants was hardly possible due to the nature of the intervention and was judged as an unclear risk (in 3 studies), high (in 2 studies). Biased rating of the measures was possible (rating scales), though not likely. Considered as serious. | Serious, downgrade one level |
| Inconsistency of results | Heterogeneity may be moderate (I^2^=42%). We judged inconsistency as borderline. | Borderline serious, do not downgrade |
| Indirectness of evidence | The study population across studies varied in diagnoses and age. The mean age of the patients across all three studies was approximately 80 years, but the studies that reported ranges in age also reported on a wide range (34-95 years). The research question was for any diagnosis and adults ≥18 years. Therefore, outcome evidence from the included studies may be applicable on the population under investigation. The intervention of one study [53] (virtual predischarge home assessment with additional access visit, if required) differed somewhat from the other studies (predischarge-home assessment visit) and might differ from current practice. However, the intense use of technological advancement in occupational therapy is expected. No systematic differences in different countries or for different clinical settings could be detected. Therefore, we had no serious concerns about the directness of evidence with respect to the interventions. Outcome measures are judged as highly patient-important and were therefore seen as direct. The comparator in all of the studies was usual care, but usual care was not always described in detail and differs in different countries. This may possibly affect the certainty. This was judged as borderline serious. | Borderline serious, do not downgrade |
| Imprecision | The total number of participants of 263 is less than the required OIS of 400. Appreciable harm and benefit were included. We had serious concerns regarding imprecision. | Serious, downgrade two levels |
| Likelihood of publication bias | Our literature search was comprehensive, there were no unpublished studies in the registries, and the studies reported both positive and negative outcomes. Therefore, we had no serious concerns. | Not serious |
| Overall judgment |  | ⨁OOO  Very low |
| ***Outcome: Mobility*** | | |
| Limitations in the design and implementation of available studies suggesting high likelihood of bias** | Two trials [29, 53] reported on mobility with low risk of bias. In both studies, blinding of personnel and participants was hardly possible due to the nature of the intervention. One study was judged as an unclear risk, the other one had low risk. Biased rating of the measures was possible (patient-reported) in one study, though not likely. Considered as borderline serious. | Borderline serious, do not downgrade |
| Inconsistency of results | The statistical heterogeneity may be substantial (I^2^=78%). Overall, the inconsistency may be substantial. | Serious,  downgrade one level |
| Indirectness of evidence | The study population across studies varied in diagnoses and age. The mean age of the patients across all three studies was approximately 80 years, but the studies that reported ranges in age also reported on a wide range (38-86 years). The research question was for any diagnosis and adults ≥18 years. Therefore, the outcome evidence from the included studies may be applicable on the population under investigation. The intervention of one study [11] (virtual predischarge home assessment with additional access visit, if required) differed somewhat from the other studies (predischarge-home assessment visit) and might differ from current practice. However, the intense use of technological advancement in occupational therapy is expected. No systematic differences in different countries or for different clinical settings could be detected. Therefore, we had no serious concerns about the directness of evidence with respect to the interventions. Outcome measures are judged as highly patient-important and were therefore seen as direct. The comparator in all of the studies was usual care, but usual care was not always described in detail and differs in different countries. This may possibly affect the certainty. This was judged as borderline serious. | Borderline serious, do not downgrade |
| Imprecision | The total number of participants of 26 is far less than the required OIS of 400. Appreciable harm and benefit were included in pooled effect. We had serious concerns regarding imprecision. | Serious, downgrade two levels |
| Likelihood of publication bias | Our literature search was comprehensive, there were no unpublished studies in the registries, and the studies reported both positive and negative outcomes. Therefore, we had no serious concerns. | Not serious |
| Overall judgment |  | ⨁OOO  Very low |
| ***Outcome: fear of falling*** | | |
| Limitations in the design and implementation of available studies suggesting high likelihood of bias** | Three trials [28, 36, 53] reported on fear of falling with low risk of bias. In all studies, blinding of personnel and participants was hardly possible due to the nature of the intervention and was judged as an unclear risk in one and high risk in the other study. Biased rating of the measures was possible (patient reported), though not likely. Considered as borderline serious. | Serious, downgrade one level |
| Inconsistency of results | Statistical heterogeneity may be moderate (I^2^=51%). No serious concerns for inconsistency. | Borderline serious, do not downgrade |
| Indirectness of evidence | The patients in the studies were comparable. The interventions of both studies differed somewhat (virtual predischarge home assessment and predischarge-home assessment by occupational therapist and patient), and in one study [10] the comparator group sometimes received more intensive treatment. Therefore, we judged indirectness as borderline. | Borderline serious, do not downgrade |
| Imprecision | The total number of participants of 85 is far less than the required OIS of 400. Appreciable harm and benefit were included in the pooled effect. We had serious concerns regarding imprecision. | Serious, downgrade two levels |
| Likelihood of publication bias | Our literature search was comprehensive, there were no unpublished studies in the registries, and the studies reported both positive and negative outcomes. Therefore, we had no serious concerns. | Not serious |
| Overall judgment |  | ⨁OOO  Very low |
| ***Outcome: Risk of Falling*** | | |
| Limitations in the design and implementation of available studies suggesting high likelihood of bias** | Five studies [16, 29, 34, 35, 39, 43] reported on risk of falling. In all of the studies, blinding of personnel and participants was hardly possible due to the nature of the intervention but it was unlikely that blinding affected this clinical outcome. Therefore, we judged a low risk. Biased counting of falls was possible but may have occurred equally in both the control and intervention groups. Considered as not serious. | Not serious |
| Inconsistency of results | Statistical heterogeneity may be low (I^2^=0%). No serious concerns for inconsistency. | Not serious |
| Indirectness of evidence | The study population across studies varied in diagnoses and age. The mean age of the patients across all three studies was approximately 80 years, but the studies that reported ranges in age also reported on a wide range (34 to 88 years). The research question was for any diagnosis and adults ≥18 years. Therefore, the outcome evidence from the included studies may be applicable on the population under investigation. The intervention of one study [53] (virtual predischarge home assessment with additional access visit, if required) differed somewhat from the other studies (predischarge-home assessment visit) and might differ from current practice. However, the intense use of technological advancement in occupational therapy is expected. No systematic differences in different countries or for different clinical settings could be detected. Therefore, we had no serious concerns about the directness of evidence with respect to the interventions. Outcome measures are judged as highly patient-important and were therefore seen as direct. The comparator in all of the studies was usual care, but usual care was not always described in detail and differs in different countries. This may possibly affect the certainty and was judged as borderline serious. | Borderline serious, do not downgrade |
| Imprecision | The total number of study participants of 501 lies above the threshold regarding the optimal information size (OIS) of 400.  Appreciable harm and benefit were included in the confidence intervals of all studies. Therefore, we had some concerns regarding imprecision. | Serious, downgrade one level |
| Likelihood of publication bias | Our literature search was comprehensive, there were no unpublished studies in registries, and the studies reported both positive and negative outcomes. Therefore, we had no serious concerns. | Not serious |
| Overall judgment |  | ⨁⨁⨁O  Moderate |
| ***Outcome: Risk of readmission*** | | |
| Limitations in the design and implementation of available studies suggesting high likelihood of bias** | Five studies [10, 16, 29, 34, 43] reported on risk of readmission. In all of the studies, blinding of personnel and participants was hardly possible due to the nature of the intervention, but it was unlikely that blinding affected this clinical outcome. Therefore, we judged a low risk. Biased counting of readmissions was possible but may have occurred equally in both the control and intervention groups. Considered as not serious. | Not serious |
| Inconsistency of results | Statistical heterogeneity may be moderate (I^2^=43%). No serious concerns for inconsistency. | Not serious |
| Indirectness of evidence | Patients and interventions in studies were comparable. Judged as not serious. | Not serious |
| Imprecision | The total number of study participants of 590 lies above the threshold regarding the optimal information size (OIS) of 400.  Appreciable harm and benefit were included in the confidence intervals of all studies. Therefore, we had some concerns regarding imprecision. | Serious, downgrade one level |
| Likelihood of publication bias | Our literature search was comprehensive, there were no unpublished studies in the registries, and the studies reported both positive and negative outcomes. Therefore, we had no serious concerns. | Not serious |
| Overall judgment |  | ⨁⨁⨁O  Moderate |

| **** Further guidelines for factor 1 (of 5) in a GRADE assessment: Going from assessments of risk of bias to judgments about study limitations for main outcomes:** | | | | |
| --- | --- | --- | --- | --- |
| **Risk of bias** | **Across studies** | **Interpretation** | **Considerations** | **GRADE assessment of study limitations** |
| Low risk of bias | Most information is from studies at low risk of bias. | Plausible bias unlikely to seriously alter the results. | No apparent limitations. | No serious limitations, do not downgrade. |
| Unclear risk of bias | Most information is from studies at low or unclear risk of bias. | Plausible bias that raises some doubt about the results. | Potential limitations are unlikely to lower confidence in the estimate of effect. | No serious limitations, do not downgrade. |
|  |  |  | Potential limitations are likely to lower confidence in the estimate of effect. | Serious limitations, downgrade one level. |
| High risk of bias | The proportion of information from studies at high risk of bias is sufficient to affect the interpretation of results. | Plausible bias that seriously weakens confidence in the results. | Crucial limitation for one criterion, or some limitations for multiple criteria, sufficient to lower confidence in the estimate of effect. | Serious limitations, downgrade one level. |
|  |  |  | Crucial limitation for one or more criteria sufficient to substantially lower confidence in the estimate of effect. | Very serious limitations, downgrade two levels. |

References

[1] Aplin, T., Jonge, D. de, and Gustafsson, L. 2013. Understanding the dimensions of home that impact on home modification decision making. *Australian occupational therapy journal* 60, 2, 101–109.

[2] Atwal, A., McIntyre, A., Craik, C., and Hunt, J. 2008. Older adults and carers' perceptions of pre-discharge occupational therapy home visits in acute care. *Age and ageing* 37, 1, 72–76.

[3] Atwal, A., Money, A., and Harvey, M. 2014. Occupational therapists' views on using a virtual reality interior design application within the pre-discharge home visit process. *Journal of medical Internet research* 16, 12, e283.

[4] Atwal, A., Spiliotopoulou, G., Plastow, N., McIntyre, A., and McKay, E. A. 2012. Older Adults' Experiences of Occupational Therapy Predischarge Home Visits: A Systematic Thematic Synthesis of Qualitative Research. *British Journal of Occupational Therapy* 75, 3, 118–127.

[5] Atwal, A., Spilliotopoulou, G., Stradden, J., Fellows, V., Anako, E., Robinson, L., and McIntyre, A. 2014. Factors influencing occupational therapy home visit practice A qualitative study. *Scandinavian Journal of Occupational Therapy,* 21, 40–47.

[6] Barberger-Gateau, P., Dartigues, J. F., and Letenneur, L. 1993. Four Instrumental Activities of Daily Living Score as a predictor of one-year incident dementia. *Age and ageing* 22, 6, 457–463.

[7] Braun, V. and Clarke, V. 2006. Using thematic analysis in psychology. *Qualitative Research in Psychology* 3, 2, 77–101.

[8] Brorsson, B., Ifver, J., and Hays, R. D. 1993. The Swedish Health-Related Quality of Life Survey (SWED-QUAL). *Quality of life research : an international journal of quality of life aspects of treatment, care and rehabilitation* 2, 1, 33–45.

[9] Cameron, J. I., Bastawrous, M., Marsella, A., Forde, S., Smale, L., Friedland, J., Richardson, D., and Naglie, G. 2014. Stroke survivors', caregivers', and health care professionals' perspectives on the weekend pass to facilitate transition home. *Journal of rehabilitation medicine* 46, 9, 858–863.

[10] Clemson, L., Lannin, N. A., Wales, K., Salkeld, G., Rubenstein, L., Gitlin, L., Barris, S., Mackenzie, L., and Cameron, I. D. 2016. Occupational Therapy Predischarge Home Visits in Acute Hospital Care: A Randomized Trial. *Journal of the American Geriatrics Society* 64, 10, 2019–2026.

[11] Collen, F. M., Wade, D. T., Robb, G. F., and Bradshaw, C. M. 1991. The Rivermead Mobility Index: A further development of the Rivermead Motor Assessment. *International Disability Studies* 13, 2, 50–54.

[12] Collin, C., Wade, D. T., Davies, S., and Horne, V. 1988. The Barthel ADL Index: A reliability study. *International Disability Studies* 10, 2, 61–63.

[13] Coster, W. J. 2013. Making the best match: selecting outcome measures for clinical trials and outcome studies. *The American journal of occupational therapy : official publication of the American Occupational Therapy Association* 67, 2, 162–170.

[14] Craig, P., Dieppe, P., Macintyre, S., Michie, S., Nazareth, I., and Petticrew, M. 2008. Developing and evaluating complex interventions: the new Medical Research Council guidance. *BMJ (Clinical research ed.)* 337, a1655.

[15] *Critical Appraisal Skills Programme (CASP). Qualitative Research Checklist.* https://​casp-uk.net​/​casp-tools-checklists/​. Accessed 30 July 2019.

[16] Davis, A. J. and Mc Clure, P. 2019. An exploratory study of discharge planning home visits within an Irish context -- investigating nationwide practice and nationwide perspectives. *IR J OCCUP THER* 47, 2, 95–113.

[17] Drummond, A. E. R., Whitehead, P., Fellows, K., Sprigg, N., Sampson, C. J., Edwards, C., and Lincoln, N. B. 2013. Occupational therapy predischarge home visits for patients with a stroke (HOVIS): results of a feasibility randomized controlled trial. *Clinical rehabilitation* 27, 5, 387–397.

[18] Godfrey, M., Cornwell, P., Eames, S., Hodson, T., Thomas, T., and Gillen, A. 2019. Pre-discharge home visits: A qualitative exploration of the experience of occupational therapists and multidisciplinary stakeholders. *AUST OCCUP THER J* 66, 3, 249–257.

[19] Goldberg, D. P. and Williams, P. 1988. *The users guide to the GHQ.*

[20] Graham, J. E., Granger, C. V., Karmarkar, A. M., Deutsch, A., Niewczyk, P., DiVita, M. A., and Ottenbacher, K. J. 2014. The Uniform Data System for Medical Rehabilitation: Report of Follow-up Information on Patients Discharged from Inpatient Rehabilitation Programs in 2002 – 2010. *American journal of physical medicine & rehabilitation / Association of Academic Physiatrists* 93, 3, 231–244.

[21] Hagsten, B., Svensson, O., and Gardulf, A. 2004. Early individualized postoperative occupational therapy training in 100 patients improves ADL after hip fracture: a randomized trial. *Acta orthopaedica Scandinavica* 75, 2, 177–183.

[22] Hagsten, B., Svensson, O., and Gardulf, A. 2006. Health-related quality of life and self-reported ability concerning ADL and IADL after hip fracture: a randomized trial. *Acta orthopaedica* 77, 1, 114–119.

[23] Hebert, R., Guilbault, J., Desrosiers, J., and Debuc, N. 2001. THE FUNCTIONAL AUTONOMY MEASUREMENT SYSTEM (SMAF): A CLINICAL-BASED INSTRUMENT FOR MEASURING DISABILITIES AND HANDICAPS IN OLDER PEOPLE. *Geriatrics Today: Journal of Canadian Geriatric Society,* September, 1–7.

[24] Hibberd, J. 2008. The home‐visiting process for older people in the in‐patient intermediate care services. *Quality Ageing Older Adults* 9, 1, 13–23.

[25] Higgins, J. and Green, S. *Cochrane Handbook for Systematic Reviews of Interventions Version 5.1.0 [updated March 2011]*.

[26] Hoffmann, T. C., Glasziou, P. P., Boutron, I., Milne, R., Perera, R., Moher, D., Altman, D. G., Barbour, V., Macdonald, H., Johnston, M., Lamb, S. E., Dixon-Woods, M., McCulloch, P., Wyatt, J. C., Chan, A.-W., and Michie, S. 2014. Better reporting of interventions: template for intervention description and replication (TIDieR) checklist and guide. *BMJ (Clinical research ed.)* 348, g1687.

[27] Jette, A. M., Haley, S. M., Coster, W. J., Kooyoomjian, J. T., Levenson, S., Heeren, T., and Ashba, J. 2002. Late Life Function and Disability Instrument: I. Development and Evaluation of the Disability Component. *Journal of Gerontology: Medical Sciences* 57a, 4, M209–M216.

[28] Katz, S., Downs, T. D., Cash, H. R., and Grotz, R. C. 1970. Progress in development of the index of ADL. *The Gerontologist* 10, 1, 20–30.

[29] Klein, R. M. and Bell, B. 1982. Self-care skills: behavioral measurement with Klein-Bell ADL scale. *Archives of Physical Medicine and Rehabilitation* 63, 7, 335–338.

[30] Lannin, N. A., Clemson, L., and McCluskey, A. 2011. Survey of current pre-discharge home visiting practices of occupational therapists. *Australian occupational therapy journal* 58, 3, 172–177.

[31] Lannin, N. A., Clemson, L., McCluskey, A., Lin, C.-W. C., Cameron, I. D., and Barras, S. 2007. Feasibility and results of a randomised pilot-study of pre-discharge occupational therapy home visits. *BMC health services research* 7, 42.

[32] Law, M., Baptiste, S., McColl, M., Opzoomer, A., Polatajko, H., and Pollock, N. 1990. The Canadian occupational performance measure: an outcome measure for occupational therapy. *Canadian journal of occupational therapy. Revue canadienne d'ergotherapie* 57, 2, 82–87.

[33] Letts, L., Wilkins, S., Law, M., Stewart, d., Bosch, J., and Westmorland, M. 2007. *Guidelines for Critical Review Form: Qualitative Studies (Version 2.0)*.

[34] Liberati, A., Altman, D. G., Tetzlaff, J., Mulrow, C., Gøtzsche, P. C., Ioannidis, J. P. A., Clarke, M., Devereaux, P. J., Kleijnen, J., and Moher, D. 2009. The PRISMA statement for reporting systematic reviews and meta-analyses of studies that evaluate health care interventions: explanation and elaboration. *PLoS medicine* 6, 7, e1000100.

[35] Linacre, J. M., Heinemann, A. W., Wright, B. D., Granger, C. V., and Mamilton, B. B. 1994. The Structure and Stability of the Functional Independence Measure. *Archives of Physical Medicine and Rehabilitation,* 75.

[36] Lincoln, N. B., Sutcliffe, L. M., and Unsworth, G. Validation of the Stroke Aphasic Depression Questionnaire (SADQ) for use with patients in hospital.

[37] Lockwood, K. J., Harding, K. E., Boyd, J. N., and Taylor, N. F. 2019. Predischarge home visits after hip fracture: a randomized controlled trial. *CLIN REHABIL* 33, 4, 681–692.

[38] Lockwood, K. J., Harding, K. E., Boyd, J. N., and Taylor, N. F. 2020. Home visits by occupational therapists improve adherence to recommendations: Process evaluation of a randomised controlled trial. *AUST OCCUP THER J* 67, 4, 287–296.

[39] Lockwood, K. J., Taylor, N. F., and Harding, K. E. 2015. Pre-discharge home assessment visits in assisting patients' return to community living: A systematic review and meta-analysis. *Journal of rehabilitation medicine* 47, 4, 289–299.

[40] Money, A. G., Atwal, A., Young, K. L., Day, Y., Wilson, L., and Money, K. G. 2015. Using the Technology Acceptance Model to explore community dwelling older adults' perceptions of a 3D interior design application to facilitate pre-discharge home adaptations. *BMC medical informatics and decision making* 15, 73.

[41] Mroz, T. M., Pitonyak, J. S., Fogelberg, D., and Leland, N. E. 2015. Client Centeredness and Health Reform: Key Issues for Occupational Therapy. *The American journal of occupational therapy : official publication of the American Occupational Therapy Association* 69, 5, 6905090010p1-8.

[42] Nikolaus, T. and Bach, M. 51. Preventing Falls in Community-Dwelling Frail Older People Using a Home Intervention Team (HIT): Results From the Randomized Falls-HIT Trial. *Journal of the American Geriatrics Society* 2003, 3 (51), 300–305.

[43] Nikolaus, T., Detterbeck, H., Gartner, U., Gnielka, M., Lempp-Gast, I., Renk, C., Suck-Röhrig, U., Oster, P., and Schlierf, G. 1995. Der diagnostische Hausbesuch im Rahmen des stationären geriatrischen Assessments. *Zeitschrift fur Gerontologie und Geriatrie* 28, 1, 14–18.

[44] Nouri, F. and Lincoln, N. 1987. An extended activities of daily living scale for stroke patients. *Clinical rehabilitation,* 1, 301–305.

[45] Nygård, L., Grahn, U., Rudenhammar, A., and Hydling, S. 2004. Reflecting on practice: are home visits prior to discharge worthwhile in geriatric inpatient care? *Scandinavian journal of caring sciences* 18, 2, 193–203.

[46] Pardessus, V., Puisieux, F., Di Pompeo, C., Gaudefroy, C., Thevenon, A., and Dewailly, P. 2002. Benefits of home visits for falls and autonomy in the elderly: A randomized trial study. *American Journal of Physical Medicine & Rehabilitation,* 81, 247–252.

[47] Pena, S. B., Guimarães, H. C. Q. C. P., Lopes, J. L., Guandalini, L. S., Taminato, M., Barbosa, D. A., and Barros, A. L. B. L. d. 2019. F ear of falling and risk of falling: a systematic review and meta-analysis. *Acta paul. enferm.* 32, 4, 456–463.

[48] Provencher, V., Clemson, L., Wales, K., Cameron, I. D., Gitlin, L. N., Grenier, A., and Lannin, N. A. 2020. Supporting at-risk older adults transitioning from hospital to home: who benefits from an evidence-based patient-centered discharge planning intervention? Post-hoc analysis from a randomized trial. *BMC GERIATR* 20, 1, 84ÔÇÉ.

[49] Rabin, R. and Charro, F. de. 2001. EQ-5D: a measure of health status from the EuroQol Group. *Annals of medicine* 33, 5, 337–343.

[50] Robinson, B. C. 1983. Validation of a Caregiver Strain Index. *Journal of Gerontology* 38, 3, 344–348.

[51] Schünemann H, Brożek J, Guyatt G, Oxman A, editors. 2013. *GRADE handbook for grading quality of evidence and strength of recommendations. Updated October 2013.*

[52] Sim, S., Barr, C. J., and George, S. 2015. Comparison of equipment prescriptions in the toilet/bathroom by occupational therapists using home visits and digital photos, for patients in rehabilitation. *Australian occupational therapy journal* 62, 2, 132–140.

[53] Sterling, M. 2011. General Health Questionnaire – 28 (GHQ-28). *Journal of Physiotherapy* 57, 4, 259.

[54] The EuroQol Group. 1990. EuroQol - a new facility for the measurement of health-related quality of life. *Health Policy* 16, 3, 199–208.

[55] Thomas, J. and Harden, A. 2008. Methods for the thematic synthesis of qualitative research in systematic reviews. *BMC medical research methodology* 8, 45.

[56] Threapleton, K., Newberry, K., Sutton, G., Worthington, E., and Drummond, A. 2017. Virtually home: Exploring the potential of virtual reality to support patient discharge after stroke. *British Journal of Occupational Therapy* 80, 2, 99–107.

[57] Threapleton, K., Newberry, K., Sutton, G., Worthington, E., and Drummond, A. 2018. Virtually home: Feasibility study and pilot randomised controlled trial of a virtual reality intervention to support patient discharge after stroke. *British Journal of Occupational Therapy* 81, 4, 196–206.

[58] Tinetti, M. E. 1986. Performance-Oriented Assessment of Mobility Problems in Elderly Patients. *Journal of the American Geriatrics Society,* 34, 119–126.

[59] Tong, A., Flemming, K., McInnes, E., Oliver, S., and Craig, J. 2012. Enhancing transparency in reporting the synthesis of qualitative research: ENTREQ. *BMC medical research methodology* 12, 181.

[60] Tooth, L. R., McKenna, K. T., Smith, M., and O'Rourke, P. K. 2003. Reliability of scores between stroke patients and significant others on the Reintegration to Normal Living (RNL) Index. *Disability and rehabilitation* 25, 9, 433–440.

[61] van Swieten, J. C., Koudstaal, P. J., Visser, M. C., Schouten, H. J., and van Gijn, J. 1988. Interobserver agreement for the assessment of handicap in stroke patients. *Stroke* 19, 5, 604–607.

[62] Van Swieten, J. C., Koudstaal, P. J., Visser, M. C., Schouten, H. J. A., and van Gijn, J. 1988. Interobserver Agreement for the Assessment of Handicap in Stroke Patients. *Stroke* 19, 5, 604–607.

[63] Wales, K., Salkeld, G., Clemson, L., Lannin, N. A., Gitlin, L., Rubenstein, L., Howard, K., Howell, M., and Cameron, I. D. 2018. A trial based economic evaluation of occupational therapy discharge planning for older adults: the HOME randomized trial. *CLIN REHABIL* 32, 7, 919–929.

[64] Wan, X., Wang, W., Liu, J., and Tong, T. 2014. Estimating the sample mean and standard deviation from the sample size, median, range and/or interquartile range. *BMC medical research methodology* 14, 135.

[65] Weiss, M. E., Bobay, K., bahr, S. J., Costa, L. L., and Hughes, R. G. A Model for Hospital Discharge Preparation: From Case Management to Care Transition.

[66] Welch, A. and Lowes, S. 2005. Home Assessment Visits within the Acute Setting: a Discussion and Literature Review. *British Journal of Occupational Therapy* 68, 4, 158–164.

[67] Whitehead, P., Fellows, K., Sprigg, N., Walker, M., and Drummond, A. 2014. Who should have a pre-discharge home assessment visit after a stroke? A qualitative study of occupational therapist´s views. *British Journal of Occupational Therapy* 77, 8, 384–391.

[68] Yam, C. H. K., Wong, E. L. Y., Chan, F. W. K., Wong, F. Y. Y., Leung, M. C. M., and Yeoh, E. K. 2010. Measuring and preventing potentially avoidable hospital readmissions: a review of the literature. *Hong Kong medical journal = Xianggang yi xue za zhi* 16, 5, 383–389.

[69] Yardley, L., Beyer, N., Hauer, K., Kempen, G., Piot-Ziegler, C., and Todd, C. 2005. Development and initial validation of the Falls Efficacy Scale-International (FES-I). *Age and ageing 34, 6, 614–619.*
