# Supplementary material for: Effects, barriers and facilitators in predischarge home assessments to improve the transition of care from the inpatient care to home in adult patients: an integrative review
Source: BMC Health Serv Res. 2021 Jun 2;21:540. doi: 10.1186/s12913-021-06386-4 (PMC8170965; doi:10.1186/s12913-021-06386-4)

**Additional file 6**

***Forest plots of comparisons***

Quality of life, various scales


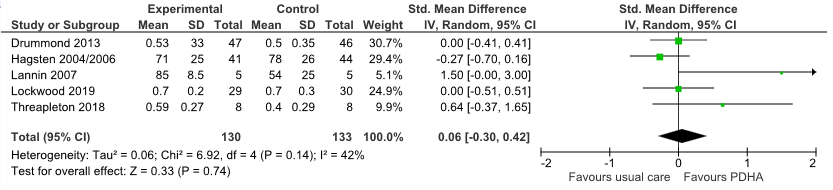


Quality of life, EQ-5D


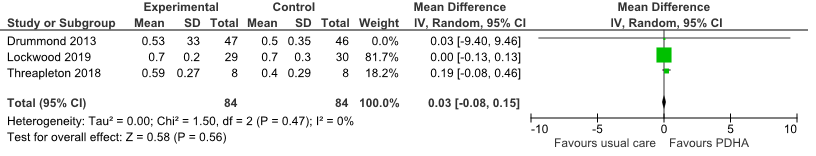


Risk of falling


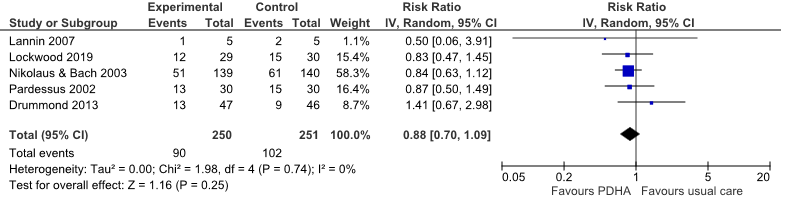


Risk of readmission


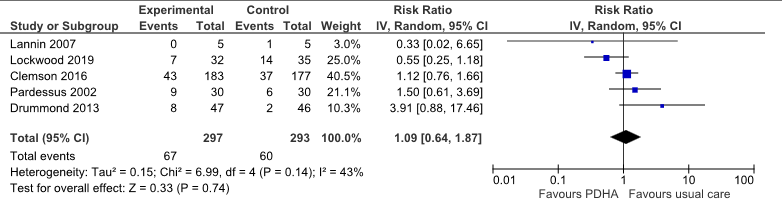


IADL, NEADL


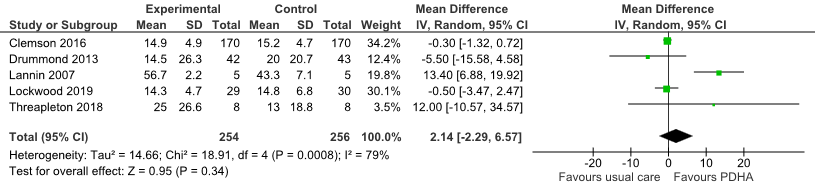


IADL/ADL, various scales


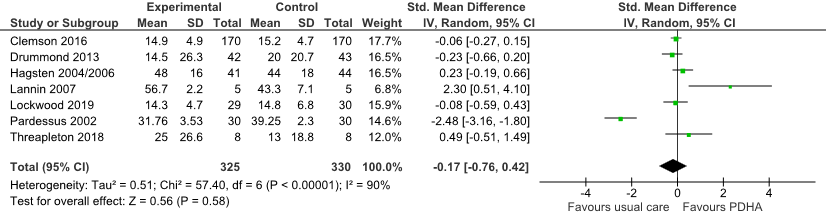


Fear of falling


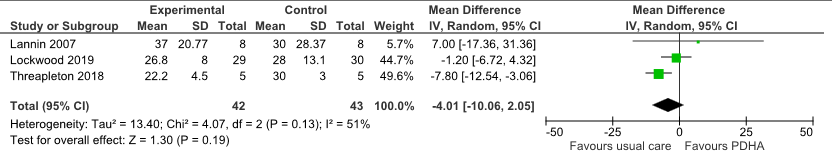


Mobility


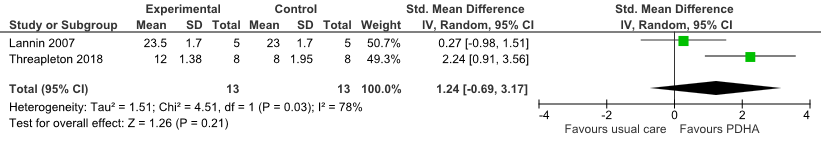

Supplement: Supplementary file 6 — Additional file 6. Forest plots. Graphical display of meta-analysis with effects in all pooled outcomes. [file 12913_2021_6386_MOESM6_ESM.docx]
