# Supplementary material for: Effects, barriers and facilitators in predischarge home assessments to improve the transition of care from the inpatient care to home in adult patients: an integrative review
Source: BMC Health Serv Res. 2021 Jun 2;21:540. doi: 10.1186/s12913-021-06386-4 (PMC8170965; doi:10.1186/s12913-021-06386-4)
Supplement: Supplementary file 7 — Additional file 7. Descriptive themes. Summary of findings in qualitative studies on level of descriptive themes. [file 12913_2021_6386_MOESM7_ESM.docx]

**Additional File 7**

| **Summary of findings in qualitative studies- descriptive themes** | | | | |
| --- | --- | --- | --- | --- |
| 1. **Rationales for conducting PDHA – When , why and which kind of PDHA** | | | | |
| **General value of PDHA** | **Therapists` satisfaction with PDHA** | | | - In general, therapists valued PDHA as beneficial to enhance a safe patient discharge in terms of identifying hazards or barriers to independence and therefore minimizing risk if falling and readmission and maximizing participation and safety [5, 6, 9]. - Furthermore OTs assumed that their patients will benefit by strengthening reassurance, confidence and independence [5, 6]. - Therapists could gain confidence with regard to the discharge destination [6]. |
|  | **Patients` and carers` satisfaction** | | | - Studies explored different satisfaction experiences in patients: - Some patients were satisfied [7]. Carers found PDHA helpful [1]. - Some patients were only partly satisfied, caused by alternative perceptions of problems and solutions [9] |
| **Contextual and pragmatic  influences** | **Setting dynamics** | | | - Differences between acute and subacute hospitals: Referrals only, if discharge destination is not home, PDHA more likely in rehabilitation setting [6] - Discharge destination is out of hospital catchment zone [6] - Full filling formal requirements for PDHA [6] |
|  | **Resource availability** | | | - Staffing level and time [6] - Resource limitation causes triage in allocation of PDHA [7] - Availability of cars - Availability and collaboration of /with community services and care support [6, 11] - Supportive social network: The necessity of a home visit could be influenced by an intact social network and the possible family support. A home visit could be unnecessary if a community or follow-up service was in charge or family members were active [10, 12]. Conversely, for Weekend Passes, the family support was important, especially if no community care services were available or recommended [5]. [9, 11] versus patient living alone with minimal support [5] |
|  | **Home visit as the only source**  **of information** | | | - A home visit could be indicated if there is no clear and proven information about the patient’s home and possible risk factors [12]. |
|  | **patient/family/carer whish** | | | - Sometimes home visits were overridden by other factors such as the explicit wish of the patients or concerns of their families [2, 4, 12]. |
|  | **“Gut feeling”** | | | - Sometimes there were no objective reasons, and the practical experience and the “gut feeling” prompted the OT to conduct a home visit [12]. |
| **Clinical factors** | **Level of impairment** | | | - The level of impairment was a main factor for conducting a home visit [12]. - Patients with a moderate level of impairment were seen as most eligible for delivering a home visit [12]. If OTs had doubt in the patients´ ability to manage tasks [10, 12], though the patient´s impairment and limitation in mobility were not too severe or too little [10], OTs only discussed decisions about the “right” level of impairment. Family and patients did not discuss time point or appropriateness for WPs [5]. - In particular for Weekend Passes, a certain level of mobility and medical stability was seen by OTs as required [5]. - A home visit should be conducted for patients with new and complex needs [4], specially with change in function prior to admission [6, 11]. - Function prior to admission including fall history without further explanation were mentioned as indication for PDHA [6]. - prolonged hospital admission [5] |
|  | **Kind of impairment** | | | - Cognitive impairment was partly seen as an indication for PDHA interventions:   - Cognitive issues as indication for PDHA: OTs were willing to use VR with a wide range of patients, though not for patients with visual, cognitive or perceptual deficits [10].   - PDHA, if cognition is a risk factor for injury/death [5]   - Home visits might facilitate patients’ performance during assessment [3], though OTs were unsure to determine the need for home visits and were worried that their cognitive impairment might not enable the patient to understand the nature of home visits [11]. - Perceptual problems were seen as indicators for a home visit [11]. - Physical problems were the major reason to conduct home visits [9, 11]. - Mental health problems: Access visits for patients with problems could be upsetting and were only rarely used [3, 11]. - Knee and hip replacements were indications to conduct access visits [3]. - Stroke survivors could engage with 3D applications [10]. |
|  | **Age and computer literacy (Virtual assessment only)** | | | - Virtual assessment apps were suspected to be suited more to younger people and patients with computer knowledge [11] |
| 1. **PDHA process – Intervention components and daily issues** | | | | |
| **Patient information** | - Different staff members informed patients of an occurring home visit [1]. - Short-term information about timing [1] so that patients did not feel sufficiently prepared [4]. - Patients felt partially not well enough informed before the home visits (causes anxiety) [3]. - Patients wished/valued written information [1, 4, 7]. - Lack of information during the home visit caused feelings of exclusion [1]. - Lack of information from OT to patients about the outcome of the home visit made patients feel unsure [1]. | | | |
| **Patients’ consent** | - In general, home assessments were not conducted without patients’ written or oral consent [4]. - Difficult for access visits in mental health services [4]. - Therapists and patients sometimes felt a lack of informed choice [2, 4]. | | | |
| **Requirements before PDHA** | - In case of Weekend Passes [4]   - Mental preparation and education of patients and family carers     - Patients and carers emphasized: safety in home, in social and recreational activities (e.g., dining out) and everyday activities, independent vs. assisted activities, general rules.     - Family carers wanted to be trained and prepared before discharge to feel confident to ensure safety, OTs felt the same.     - Most patients felt prepared.     - Health care providers faced challenges in preparing and educating families: difficult to understand scope and importance of WP, family was too overwhelmed to accept training.     - “Learning by doing” occurs for family carers with subsequent WPs [5].   - Aids and devices in place before conducting PDHA   - Professional care service organized, family carers are available respectively   - The readiness of the patient is judged by health care provider team in hospital (medical stabile, mobility)   - Environment is assessed to be safe and suitable for patient - in case of home visit [6]   - Co-ordination of tasks and resources: car availability, optimal time point, therapist availability, family/carer availability | | | |
| **Attendants** | - Other healthcare staff attended if the OT thought it was needed [3]. - In-hospital preparation of WPs, and OTs collaborated with other healthcare staff [4]. - Client, therapist and other healthcare staff member [3]. - Family members in particular, in case of cognitive impairment [3]. - Too many attendants caused chaos [3] and overwhelmed older people [7]. - Presence of the patient facilitated the discharge process [3]. - Same therapist for the home visit and clinic assessment was beneficial and important for cognitively impaired patients [7]. - Same therapist for the follow-up not important [9]. - Patients without cognitive impairments could decide who should be present [3]. - No attendants possible in access visits [3]. | | | |
| **Evaluation** | **Follow ups** | | - Follow up after discharge of patient was not regularly conducted, but was felt by OTs to be needed [6, 9] | |
|  | **Outcomes of PDHA from OT view** | | - Patient and carer have increased insight into care needs and functional deficits [5] - Patient and family have increased confidence [5] - Patient and carer have increased risk awareness (as a result of risk education) [5] - OT s identify non-suitability of PDHA instead of conducting PDHA [5] - “Sustainable” and “safe” discharge home [5] | |
|  | **Outcome communication** | | - Immediate outcome of PDHA is not always communicated to patients which could make them feel excluded and unsure about consequennces [1] - In case of weekend passes, outcome of “self assessment” of patient and carers during the weekend is not formally communicated to therapists- missed opportunity to reflect [4] | |
| **Issues in process** | **Patients adjustment** | | - difficulties adjusting to life outside while PDHA [2], | |
|  | **Patient involvement** | | - sometimes excluded from carers view [2] | |
|  | **Optimal timeframe [6]** | | - to short from patients view [1, 7] - to short or last minute from therapists view [3] - OTs usually needed one hour for a visit but tried to take time according to patients’ needs [4]. - Access visit was a possibility for a “last-minute” home assessment, but no chance to include the family. No attendants in access visits could save time, but not if OT talked with family members during the visit [4]. - No time pressures in mental health services [4]. - Organizational factors determined the timing of the home visit, given the time frame “of a couple of days is a luxury” [4]. - OTs would complete more visits if they had more time [12]. - Decisions were made quickly after the home visit [5]. | |
|  | **Risks while PDHA** | | - For OT: dangerous situations including potential hazards through aggressive or dangerous persons and animals, poor repair and poor hygiene of the home [5] - For patients: medical emergencies or non-compliance (not willing to go back to hospital) [5] | |
| **Potential for process improvement** | **Early patient identification** | | - “figure out the groups of people for whom they provide the most benefit” [6] - “A decision-making support tool to prompt and guide  decision-making, and enhance communication was  therefore viewed positively…”, especially for less experienced therapists [6] | |
|  | **standardized protocols** | | - standardised guidelines or protocols would afford consistency in practice during visits, and minimize unnecessary “just in-case home visits” [5] | |
|  | **collaboration with community services** | | - Occupational Therapists, public health nurses and other community base teams to maximize sustainability of home discharges [5] | |
|  | **resources** | | - Organizational backup [5] - OT assistants while PDHA [5] - Designated cars/timely taxis [5] - More time for OT assessment and for the patient to adjust while PDHA [5] - VR solutions were seen by therapists to potentially reduce time and resources required for agreements regarding modifications and aids [2] | |
|  | **Use of virtual reality applications for environmental assessment** | | - Applications were seen as preferable to 2D tools such as photographs and hand drawings and conveyed additional information [3]. - OTs and patients could imagine the use across a range of scenarios and clinical fields [3, 9], except for patients with cognitive impairment [3]. - Some patients were already familiar with 3D apps for visualization from other areas such as real estate business and furniture shops [9]. - 3D tool was partly seen as usable for patients’ independent environmental assessment [3], but in regard to implementing special OT equipment, special OT knowledge and supervision were needed [9]. - Application was felt to be rather useful for major modifications [3, 9]. - Use of virtual applications was felt to reduce time for negotiation and implication of modifications [3]. - Some OTs worried that the IT system and technical resources of hospital wards were too restricted for use [11], that apps threaten a central aspect of OT [3, 11] and that there was greater uncertainty about ensuring that appropriate action would be taken after the assessment [11]. - Some OTs found it easy to learn to operate the app [3], while some found it time consuming [3, 9]. | |
| 1. ***Balancing abilities and environment for safe discharge [3, 11]***   *“They [Occupational Therapists] appeared to balance information about the person with information about the home environment (the physical characteristics of the home and the availability of care support), in order to ascertain whether the two were compatible.” [11]* | | | | |
| **3.1 Functional patient assessment in the home environment** | | | | |
| - To check, if the patient is able to be transferred safely home from functional point of view [11] - To replicate patients functional abilities within the home environment; not possible while virtual PDHA [10], - can not be simulated in hospital [5] - Serves as continuous discharge preparation [4] | | | | |
| **Effects of presence of patient during home assessment visit** | | **Realistic view on function for OTs** [1, 3, 5, 9, 10] | | - A great advantage of an assessment in their home was to see the patient performing in their home and real life setting [3, 5, 9]. - Possibility of assessment of mental and cognitive state [3]. - A clinic assessment only could not reveal the potential problems and risks [3, 5, 9, 11]. - There is a potential risk in the lack of validity of the abilities assessment because the patients may have been extra careful during the home visit [4]. - Sometimes supposed risks in follow-up were not confirmed [10], and sometimes patients’ needs changed after discharge. Follow-ups were felt as essential by OTs to discover unrecognized and new needs [10]. |
|  |  | **Opportunity for patients` self -assessment** | | - Gave the patient a self-assessment and realistic view of their own capabilities[4, 10, 12], helped to accept assistance [10], demonstrated family carers’ difficulties to fulfill complex patient needs [12]. |
|  |  | **Influence on feelings and motivation of patients** | | - The home visit could trigger different emotions as a result of stress in the testing-situation [2, 7] or positive or negative feedback on the performance [2]. OTs felt that the stressors could sometimes outweigh the benefits [11]. - PDHA can reduce anxiety regarding discharge [5] - Home visits could take patients fear to return home [2, 3] and some patients felt relaxed while visit [7]. They also saw it as an opportunity to see loved ones and the home and increased their wish to return home [3]. - Effects on patient motivation were described as derived from contact with loved ones and pets and just seeing the home as positive motivation [3] and as *“light at the end of the tunnel”* [6]eing unsatisfied with motor capabilities as negative motivations [9], but also could increase motivation to engage in inpatient therapy [4]. - For Weekend Passes, mixed feelings for patients and family carers were described. Patients felt excitement, nervousness and anxiety before the first WP. During the WP, some patients enjoyed the break and time with families, but some patients were overwhelmed with auditory and visual stimulation. Explicit fears concerned safety, falling, having another stroke, being expected too much. Leaving home after the WP was often sad for patients.[4] Family carers primarily had negative emotions of anxiety and having the burden of caring. They felt strongly relieved with help from neighbors and Health Care Providers. However, they also felt happiness to have the patient home [4]. |
|  |  | **Information on future inpatient therapy sessions** | | - One study [4] on Weekend Passes described that home visits can inform future inpatient therapy sessions and address difficulties experienced during the home visit. The patients could apply their skills in a real-world condition over a longer period of time. - A PDHA with patient attended might help patients to target the rehabilitation [6]. |
| **3.2 Environmental assessment** | | | | |
| - To identify needed provisions and adaptions and to o see whether patients were limited by their home environment was a substantial aim of home visits [4]. - give a picture of the home environment and thus enable practical recommendations [5] | | | | |
| **hazards identification** | | - To discuss and eliminate potential hazards for a fall [4, 5, 12], in preparation of a WP [5] before the discharge or the actual discharge [4]. - Access visits without the patient were useful to assess the home environment [4], but not the patients’ abilities. This was also true for virtual home visits [11, 12]. - Virtual PDHA can help to identify hazards and serve as educational tool [10] | | |
| **aids identification and adaptation** | |  | | - Important goal of PDHA is the adequate provision and adaptation of aids and home modifications [3, 11] - Access visits were used for this purpose [3] |
| **3.3 Home modifications and aids** | | | | |
| **Patients’ acceptance on home modifications and aids** | | **Cooperation with service providers** | | - Patients were often dissatisfied regarding the cooperation and results of the service providers. Problems included failure to transfer information to the contributor, delay in delivery of the prescribed aids or the aids had not been installed until the patient returned home, or the size of the aid was not correct [9]. In addition, the number of additional people entering the patient’s home might overwhelm, which could lead to refusal of services [9]. - 3D applications using correct measures could enhance the collaboration with service providers and reduce the number of people entering the patient’s home [2]. |
|  |  | **Patients’ own solutions for aids** | | - Some patients made alternative solutions, which could mean individually tailored adaptions, rearrangement of the placement, refraining from certain occupations or temporary adaptions of their own [9]. |
|  |  | **Patients’ attitudes and concerns regarding home modifications/aids** | | - Some patients resisted any changes and resigned recommendations or alter interventions and put themselves at risk [2, 9]. - Some patients felt that too many changes were made at the predischarge home visit [1]. - Sometimes patients they felt limited in their usual behaviors [1]. - Early imagination with VR could reduce anxiety and reserves against modifications [2]. |
|  |  | **Patients’ imagination and understanding of home modifications and aids** | | - Virtual visualization could improve the patient’s imagination of technical aids, changes and modification and the feasibility of implementation [2, 10]. It also helped them to understand why recommendations were made and to accept them [2, 10]. VR gave a clear visual of proposed changes [10]. - With VR, he immediate feedback of patients could improve the decision-making process [2]. |
|  |  | **Equal discussion and shared decision making** | | - VR could be a possibility to empower patients and give them a greater influence in the process of implementing modifications[2, 10]. The visualization could prompt patients’ memories of the home environment, which served as a draft for shared understanding and could facilitate discussions about changes[2, 10]. - more visually focused communication tools, such as VRIDAs, are likely to provide the opportunity to overcome some of the communication imbalances that exist in current practice settings [2] |

References

[1] Atwal, A., McIntyre, A., Craik, C., and Hunt, J. 2008. Older adults and carers' perceptions of pre-discharge occupational therapy home visits in acute care. *Age and ageing* 37, 1, 72–76.

[2] Atwal, A., Money, A., and Harvey, M. 2014. Occupational therapists' views on using a virtual reality interior design application within the pre-discharge home visit process. *Journal of medical Internet research* 16, 12, e283.

[3] Atwal, A., Spilliotopoulou, G., Stradden, J., Fellows, V., Anako, E., Robinson, L., and McIntyre, A. 2014. Factors influencing occupational therapy home visit practice A qualitative study. *Scandinavian Journal of Occupational Therapy,* 21, 40–47.

[4] Cameron, J. I., Bastawrous, M., Marsella, A., Forde, S., Smale, L., Friedland, J., Richardson, D., and Naglie, G. 2014. Stroke survivors', caregivers', and health care professionals' perspectives on the weekend pass to facilitate transition home. *Journal of rehabilitation medicine* 46, 9, 858–863.

[5] Davis, A. J. and Mc Clure, P. 2019. An exploratory study of discharge planning home visits within an Irish context -- investigating nationwide practice and nationwide perspectives. *IR J OCCUP THER* 47, 2, 95–113.

[6] Godfrey, M., Cornwell, P., Eames, S., Hodson, T., Thomas, T., and Gillen, A. 2019. Pre-discharge home visits: A qualitative exploration of the experience of occupational therapists and multidisciplinary stakeholders. *AUST OCCUP THER J* 66, 3, 249–257.

[7] Hibberd, J. 2008. The home‐visiting process for older people in the in‐patient intermediate care services. *Quality Ageing Older Adults* 9, 1, 13–23.

[8] Money, A. G., Atwal, A., Young, K. L., Day, Y., Wilson, L., and Money, K. G. 2015. Using the Technology Acceptance Model to explore community dwelling older adults' perceptions of a 3D interior design application to facilitate pre-discharge home adaptations. *BMC medical informatics and decision making* 15, 73.

[9] Nygård, L., Grahn, U., Rudenhammar, A., and Hydling, S. 2004. Reflecting on practice: are home visits prior to discharge worthwhile in geriatric inpatient care? *Scandinavian journal of caring sciences* 18, 2, 193–203.

[10] Threapleton, K., Newberry, K., Sutton, G., Worthington, E., and Drummond, A. 2017. Virtually home: Exploring the potential of virtual reality to support patient discharge after stroke. *British Journal of Occupational Therapy* 80, 2, 99–107.

[11] Whitehead, P., Fellows, K., Sprigg, N., Walker, M., and Drummond, A. 2014. Who should have a pre-discharge home assessment visit after a stroke? A qualitative study of occupational therapist´s views. *British Journal of Occupational Therapy* 77, 8, 384–391.
