# Supplementary material for: Effects, barriers and facilitators in predischarge home assessments to improve the transition of care from the inpatient care to home in adult patients: an integrative review
Source: BMC Health Serv Res. 2021 Jun 2;21:540. doi: 10.1186/s12913-021-06386-4 (PMC8170965; doi:10.1186/s12913-021-06386-4)
Supplement: Supplementary file 8 — Additional file 8. Analytical themes. Summary of analytical themes from qualitative synthesis with related barriers and facilitators and derived implications. [file 12913_2021_6386_MOESM8_ESM.docx]

| ***vorläufige version_update*** | | | | | |
| --- | --- | --- | --- | --- | --- |
| **Analytical themes, barriers and facilitators and implications for Predischarge Home Assessment (PDHA) interventions** | | | | | |
| **#** | **Analytical theme/**  **description** | | **Barriers** | **Facilitators** | **Implications** |
| 1 | **The safety assessment of the home environment**  Discharge home was reported as an indication of a successful DPHV, with many participants citing the importance of a “sustainable” and “safe” discharge [1]. Aim was to identify any needed provisions and adaptions to go home [3] and to identify and eliminate risks within the home [1, 3, 4], as well as to assess whether home environment fits the needed equipment [4]. | |  | •identification of risk factors and patient/family’s awareness of these factors following  education [1]  •VR is useful tool to educate patients: identify and discuss risk factors, increase patients awareness [2]  •enables practical recommendations [1] | **1 Use environmental assessments together with patients to provide education about hazards** |
| 2 | F**unctional assessment of the patient at home as a reality check**  Aim of a functional assessment is to assess whether the patient is able to manage within his/her home [4]. In general, the predischarge home visit is a chance for therapists to gain a realistic view on patients functions. But this also applies vice versa: "Is making them [the patients] aware of that impact and how they might be able to overcome the problems they will encounter. [...] We do get patients who say ´oh once I´m home I´ll be fine...`, but I don`t think they`ll always appreciate the limitations they`re going to encounter." [4]. The "social skills" of OTs are important to patients while the assessment[3]. | | •Anxiety provoking, if patients view the assessment as test, they might fail [3, 4]  • virtual environmental home assessments cannot replicate patients function in the own home environment [2] | •Gives a realistic view on function at home for OTs, and insight into difficulties and abilities for patients and carers [1, 4–8]  •gives information on future in-hospital therapy-sessions and helps to target the rehab [5–7]  •to see the home, motivates patients for therapy, aiming to go back home [1, 3, 4, 6, 7]  •reduces anxiety regarding discharge [1]  •social skills of therapists to make patients feel comfortable during the assessments [9] | **2 Conduct a functional assessment that includes the living reality of the patient and helps the patient to find individual participation goals for therapy.**  **2.1 Consider potential patient anxieties regarding the assessment situation.** |
| 3 | **Intervention planning and evaluation**  Especially novice therapists struggle with aim and content of PDHA [1]. The actual timing of PDHA is highly controlled by organizational factors and pressures [4–6]. There are often pragmatic empirical aspects, like availability of supportive network, patient whish or “gut feeling” as leading decisions about conducting PDHA or not [1, 5, 7, 8]. The mere presence of community service providers sometimes led therapists to hand over the care of the patient to them. Working with community actors often led to dissatisfaction with devices [7]. A digital interface to transmit environmental information could foster the communication between different stakeholders . | | •Problems with service providers (secondary actors) [7]  •no follow-up after discharge [5] | •Clear aims [5]  •clear assessment tasks [5]  •early patient identification and planning [5]  •decision support tool [5]  •use of standard protocols during visit [1]  •formal evaluation after PDHA [6]  •Collaboration with community services [1]  •digital interface to collaborate with service providers [10] | **3 Use standardized procedures and materials to guide the PDHA process. Digital solutions could support the collaboration between hospital and community service providers.** |
| **4** | **Patient information about the home assessment procedure**  For patients the PDHA can be anxiety provocating [3, 4]. Older people are too little informed prior to a visit and are therefore insecure [4, 12]. Patients welcome general written information about PDHA [12]. Some patients and therapists feel, that it is not given a real informed choice about the assessment [3, 4]. While carer were involved by the therapists during the assessment, the patients were sometimes excluded [3]. Patients often do not feel sufficiently informed about the outcome of the assessment and are therefore uncertain about next steps and feel excluded [3, 4]. Patients also note that there are written reports and that professionals make an evaluation among each other, but this information does not reach the patient [3]. | | •Inadequate patient information about aim, process , assessment, results and further steps make unsure and anxious [3, 4] | •written information [6, 12] | **4 Provide adequate (verbal and written) patient information about aim, process, assessment, results and consequences of the predischarge home assessment.** |
| **5** | **Patients and family carers acceptance on home modifications and aids**  Some patients have concerns to be hindered in their usual way to perform ADL [3]. Beyond that, patients have own ideas and solutions for aids and modifications (e.g. to rearrange the placement of aids such as cushions to raise seating height)[7].  Patients and family carers find it difficult to imagine modifications, adaptations [2]. OTs and older people felt that the use of visualization with a 3-D interior design software application help patients to better understand assistive technologies and adaptations [10] OTs also felt, that a virtual reality tool is superior to drawings and photographs [10]. Participants felt that the OT could communicate better about modifications or even decisions against a discharge in the patients home [2]. Providing a visualization represents a chance for patients to give immediate feedback on proposed changes [10] and facilitate equal discussions [2] which allows shared decision making [10]. | | • Patients and carers cannot imagine home modifications and aids [2]  • patients have concerns, that modifications might hinder usual performance [3] | •VR helped to give a realistic visualization of possible modifications and aids [2, 10]  •VR enables equal understanding, immediate feedback enables empowers patients [2, 4]  • patients have own solutions for aids [7] | **5 Provide tailored adaptations based on shared decision making and involve explicitly patients ideas, solutions and expectations in planning home modifications.**  **5.1 Use appropriate visualization and discuss recommended aids and home modifications.** |
| **6** | **Matching PDHA and clinical patient conditions**  Therapists identified some barriers and facilitators to be considered when deciding what kind of pre-discharge home assessment shall be performed [1, 2, 4, 5, 7, 8] . Different patient conditions need different approaches to assessment.  To use a virtual pre-discharge home assessment application, physical and cognitive and expertise about OT-equipment is needed. Possible impairments of the patient may complicate the use. When using the virtual surface as a visualization and training tool for patients, sensory and visual limitations must be considered [2]. | **hip- and knee-replacement** |  | • Predischarge home assessments without patient (access visits) | **6 Tailor the intervention components and mode of delivery to patients level and kind of impairments.** |
|  |  | **kind of impairment: visual, perceptual** | •Virtual pre-discharge home assessment might not be appropriate for bad eyesight, visual impairments, cognitive impairments, low fine motor function, low computer literacy; unfamiliar with the occupational therapy objects [2] | •Predischarge home assessments with patient present [8] |  |
|  |  | **level of impairment: minimal or very severe** | •PDHA not appropriate [8] |  |  |
|  |  | **level of impairment: moderate, new complex needs, change in function** |  | •most likely to need PDHA [4, 7, 8] |  |
|  |  | **kind of impairment: cognitive** | •PDHA not appropriate higher level (patients might not understand the scope) [7, 8] | •Assessment in hospital more useful for high level [8]  •PDHA for lower level [1, 4] |  |
|  |  | **kind of impairment: mental disorders** | •Low acceptance for access visits [4] |  |  |
| **7** | **Context factors in daily routine of PDHA**  Many of the qualitative studies identified factors that, in a clinical or patient-related context, may have a beneficial or impeding effect on the decision of whether to and how to conduct PDHA. Lack of resource availability (staff, time, secretarial backup, technical resources for virtual assessment) hamper the process of organization and execution. A virtual approach to PDHA could partially overcome some of the obstacles (e.g. out of hospital catchment zone, car availability, safety requirements for allowing a home visit with patient). Factors such as risks while at home visits and the organization of appropriate PDHA attendants have an impact on the process of PDHA. | | •Risks while at home visit (e.g. medical emergency, non-compliance, dangerous situations) [1]  •resource availability (staff, cars, time, secretarial backup, technical resources for virtual assessment)[2–5, 8, 12]  •patient home out of hospital catchment zone [5]  •formal requirements for allowing a home visit [5] | •Appropriate number and relevant attendants for PDHA [4, 7]  •Virtual home assessments save travel distances [10] | **7 Consider specific context factors in PDHA-design.** |

References

1. Davis, A.J., Mc Clure, P.: An exploratory study of discharge planning home visits within an Irish context -- investigating nationwide practice and nationwide perspectives. IR J OCCUP THER **47**(2), 95–113 (2019). doi: 10.1108/IJOT-10-2018-0015

2. Threapleton, K., Newberry, K., Sutton, G., Worthington, E., Drummond, A.: Virtually home: Exploring the potential of virtual reality to support patient discharge after stroke. British Journal of Occupational Therapy **80**(2), 99–107 (2017). doi: 10.1177/0308022616657111

3. Atwal, A., McIntyre, A., Craik, C., Hunt, J.: Older adults and carers' perceptions of pre-discharge occupational therapy home visits in acute care. Age and ageing **37**(1), 72–76 (2008). doi: 10.1093/ageing/afm137

4. Atwal, A., Spilliotopoulou, G., Stradden, J., Fellows, V., Anako, E., Robinson, L., McIntyre, A.: Factors influencing occupational therapy home visit practice A qualitative study. Scandinavian Journal of Occupational Therapy(21), 40–47 (2014)

5. Godfrey, M., Cornwell, P., Eames, S., Hodson, T., Thomas, T., Gillen, A.: Pre-discharge home visits: A qualitative exploration of the experience of occupational therapists and multidisciplinary stakeholders. AUST OCCUP THER J **66**(3), 249–257 (2019). doi: 10.1111/1440-1630.12561

6. Cameron, J.I., Bastawrous, M., Marsella, A., Forde, S., Smale, L., Friedland, J., Richardson, D., Naglie, G.: Stroke survivors', caregivers', and health care professionals' perspectives on the weekend pass to facilitate transition home. Journal of rehabilitation medicine **46**(9), 858–863 (2014). doi: 10.2340/16501977-1854

7. Nygård, L., Grahn, U., Rudenhammar, A., Hydling, S.: Reflecting on practice: are home visits prior to discharge worthwhile in geriatric inpatient care? Scandinavian journal of caring sciences **18**(2), 193–203 (2004). doi: 10.1111/j.1471-6712.2004.00270.x

8. Whitehead, P., Fellows, K., Sprigg, N., Walker, M., Drummond, A.: Who should have a pre-discharge home assessment visit after a stroke? A qualitative study of occupational therapist´s views. British Journal of Occupational Therapy **77**(8), 384–391 (2014)

9. Atwal, A., Spiliotopoulou, G., Plastow, N., McIntyre, A., McKay, E.A.: Older Adults' Experiences of Occupational Therapy Predischarge Home Visits: A Systematic Thematic Synthesis of Qualitative Research. British Journal of Occupational Therapy **75**(3), 118–127 (2012). doi: 10.4276/030802212X13311219571701

10. Atwal, A., Money, A., Harvey, M.: Occupational therapists' views on using a virtual reality interior design application within the pre-discharge home visit process. Journal of medical Internet research **16**(12), e283 (2014). doi: 10.2196/jmir.3723

11. Hibberd, J.: The home‐visiting process for older people in the in‐patient intermediate care services. Quality Ageing Older Adults **9**(1), 13–23 (2008). doi: 10.1108/14717794200800003
